# Supplementary material for: An integrated model for detecting significant chromatin interactions from high-resolution Hi-C data
Source: Nat Commun. 2017 May 17;8:15454. doi: 10.1038/ncomms15454 (PMC5442359; doi:10.1038/ncomms15454)
Supplement: Supplementary Information — Supplementary Figures, Supplementary Table, and Supplementary References [file ncomms15454-s1.pdf]

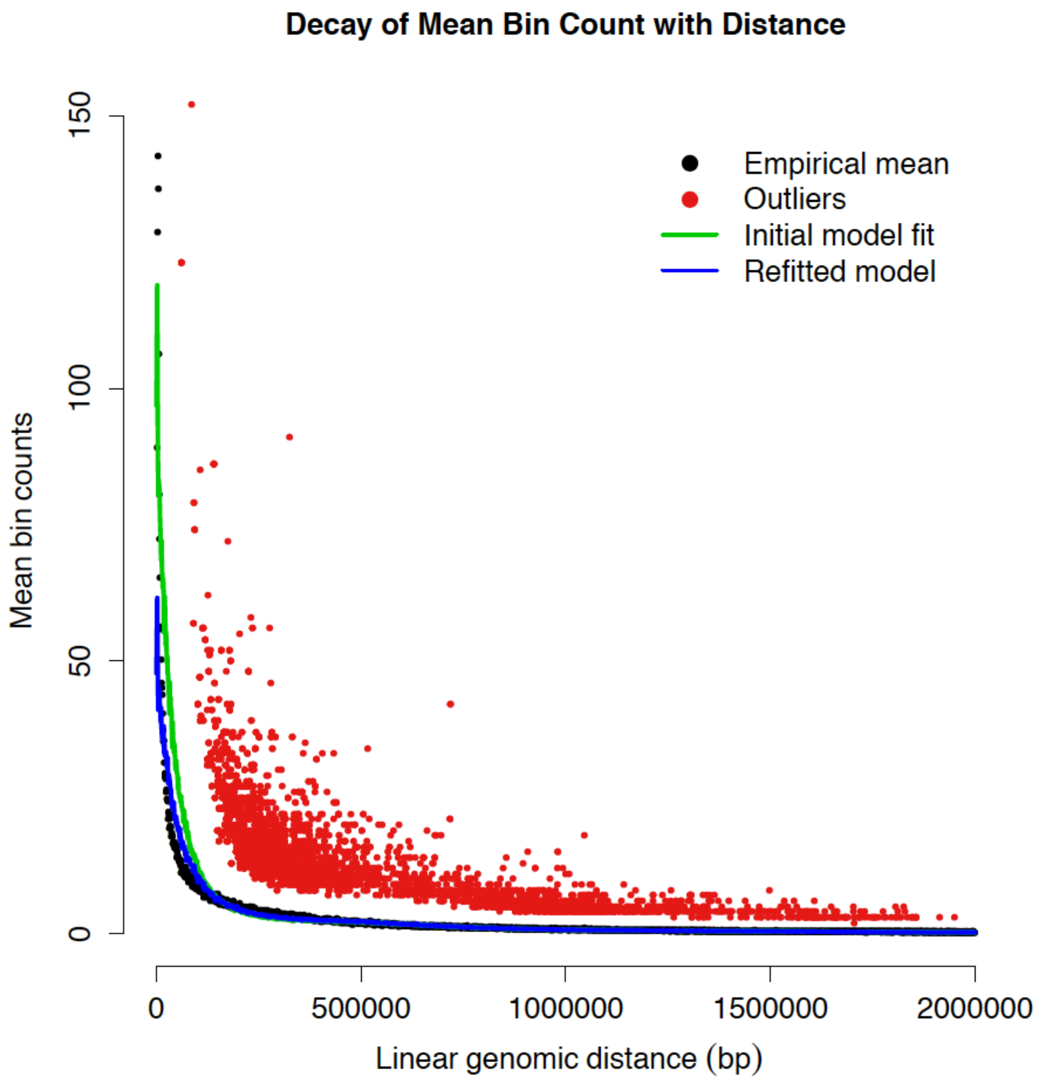

Supplementary Figure 1 – ZTNB model using hurdle regression for chromosomes 1 – X
Hurdle regression fits for the Rao et al.[1] Hi-C interaction data points sampled from chromosomes 1-X. We fit an initial model to the observed bin counts (black points) and genomic distances between all possible Hi-C pairs. The initial model fit (green) determines a threshold to identify outliers (red points), which are excluded from the calculation of a refined null represented by a second fit.

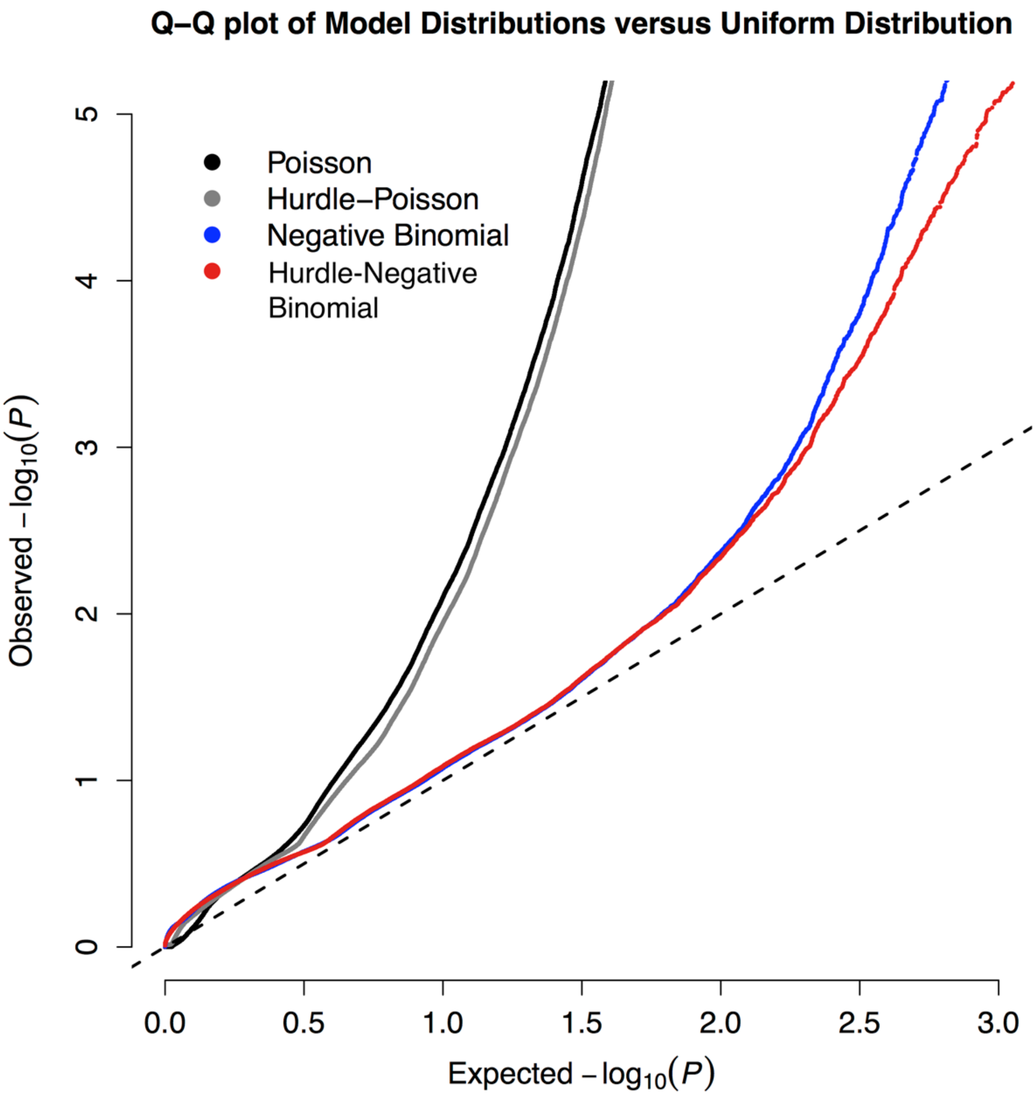

Supplementary Figure 2 – Q-Q plots of hurdle negative-binomial (ZTNB) versus Poisson, hurdle-Poisson, and negative binomial on Rao et al.[1] data

Comparison by quantile-quantile plots of Hi-C interaction data from across all chromosomes suggests that the hurdle negative binomial model (ZTNB) best accounts for both zero inflation and overdispersion, thus reducing the number of false positive interactions for a given level of significance.

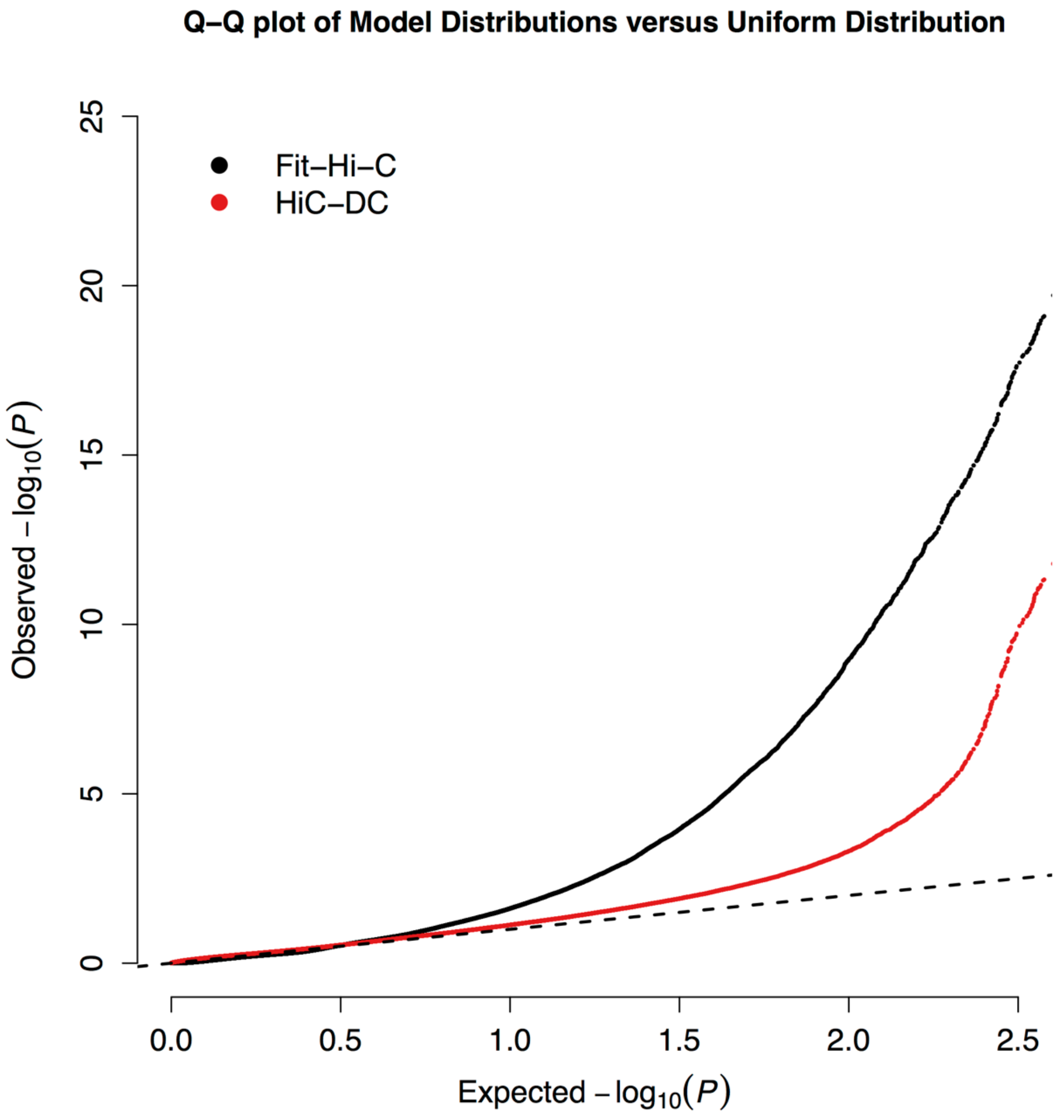

Supplementary Figure 3 – Q-Q plot of Fit-Hi-C versus HiC-DC
Quantile-quantile (Q-Q) plots of HiC-DC (red) versus Fit-Hi-C (black) on Rao et al.[1] data sampled randomly from chromosome 1 - X. Compared to *P* values drawn from the uniform distribution (dashed line), Fit-Hi-C appears to produces *P* values with inflated significance relative to HiC-DC.

a

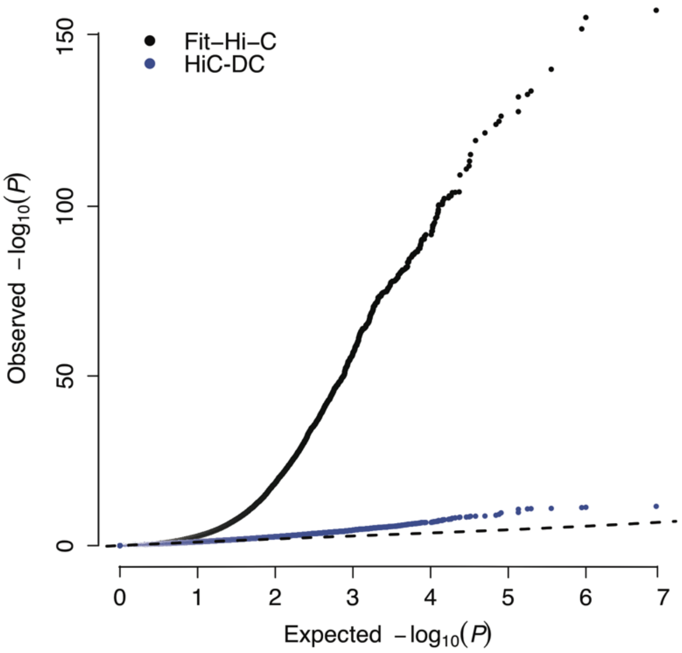

b

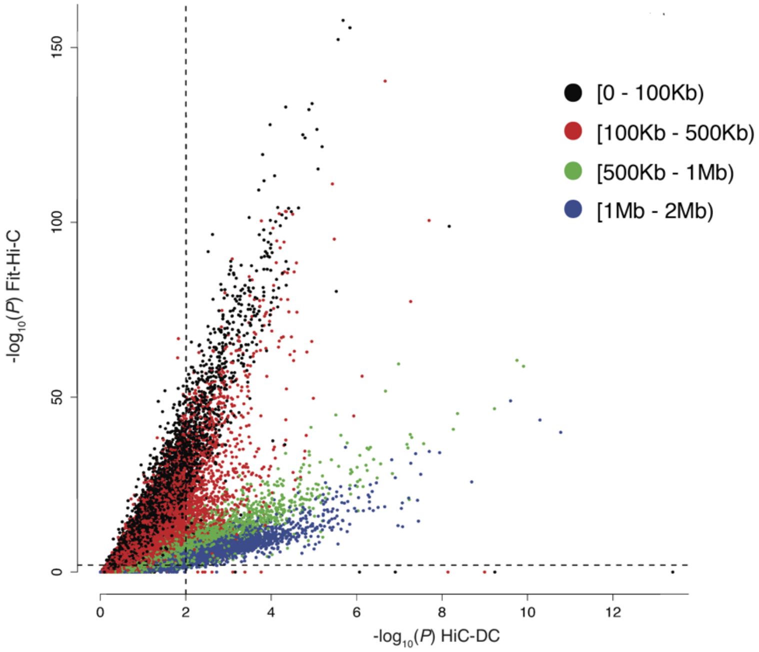

Supplementary Figure 4 – Fit-Hi-C versus HiC-DC on IMR90 data set[2]

(a) Q-Q plot of $-\log_{10} P$ values of Fit-Hi-C versus HiC-DC on Dixon et al.[2] data. Dashed line represents the uniform distribution. **(b)** Scatterplot of $-\log_{10} P$ values assigned by Fit-Hi-C versus HiC-DC on the same data, colored by contact distance. Dashed lines represent 1% FDR threshold on the $-\log_{10}$ scale for each respective method.

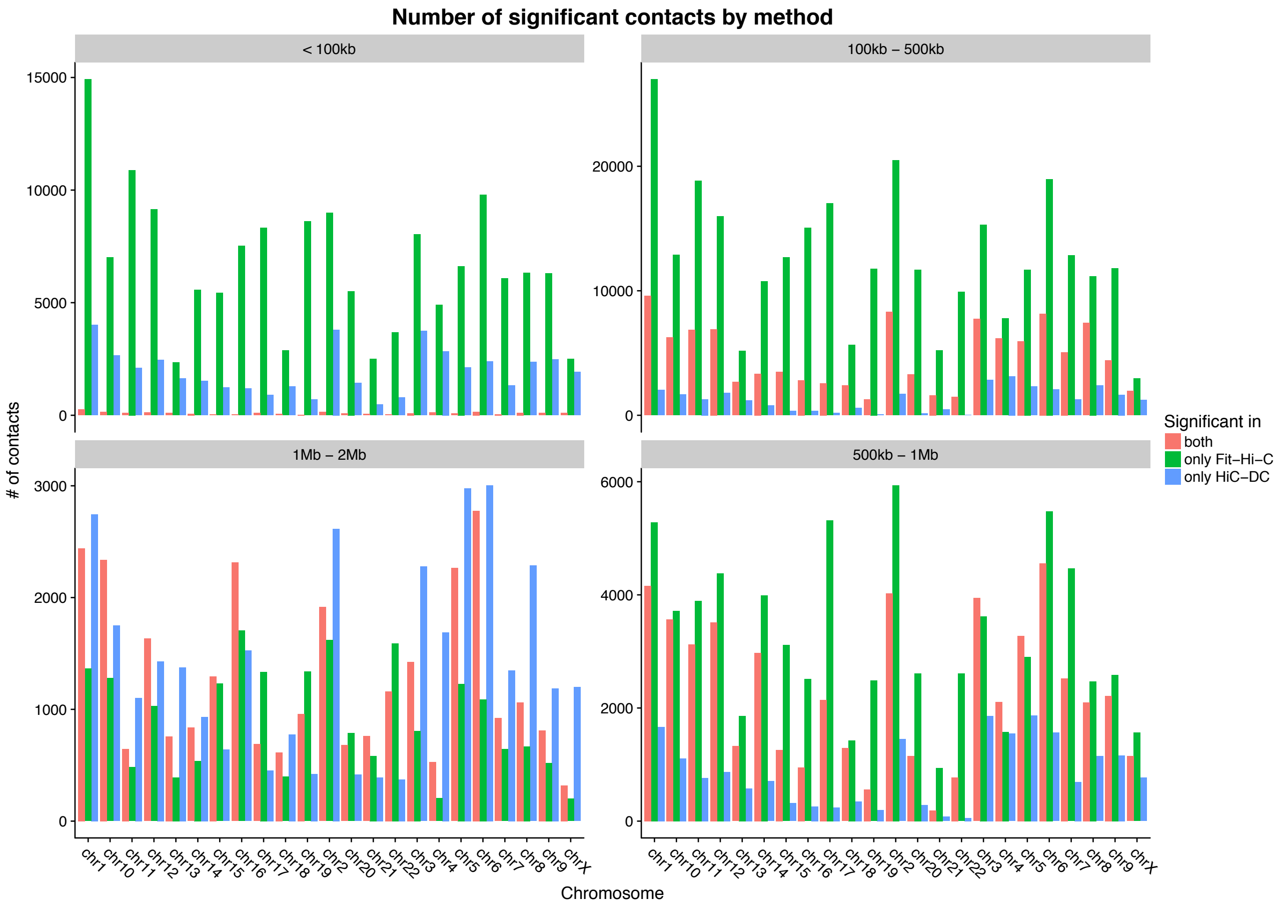

Supplementary Figure 5 – Contrasting significant contacts for both HiC-DC and Fit-Hi-C We applied HiC-DC and Fit-Hi-C to independently assign a level of significance to each contacts of the Rao et al.[1] data genome wide at the 5kb resolution, and collected the significant contacts (FDR < 1%) for each chromosome and each method. Each contact was labeled as either significant in Fit-Hi-C (but not in HiC-DC), or significant in HiC-DC (but not in Fit-Hi-C), or significant in both. We further partitioned these labeled contacts by the distance thresholds established in (**Supplementary Figure 4d**). We observed almost no agreement among significant contacts over the shortest distance, greater agreement for significant HiC-DC contacts in the 100kb – 500kb range, and the greatest agreement in the 500kb – 1Mb range. While Fit-Hi-C generally predicts more significant contacts than HiC-DC, this is reversed in longer for contacts spanning 1Mb – 2Mb. Data is reported in **Supplementary Data 1**.

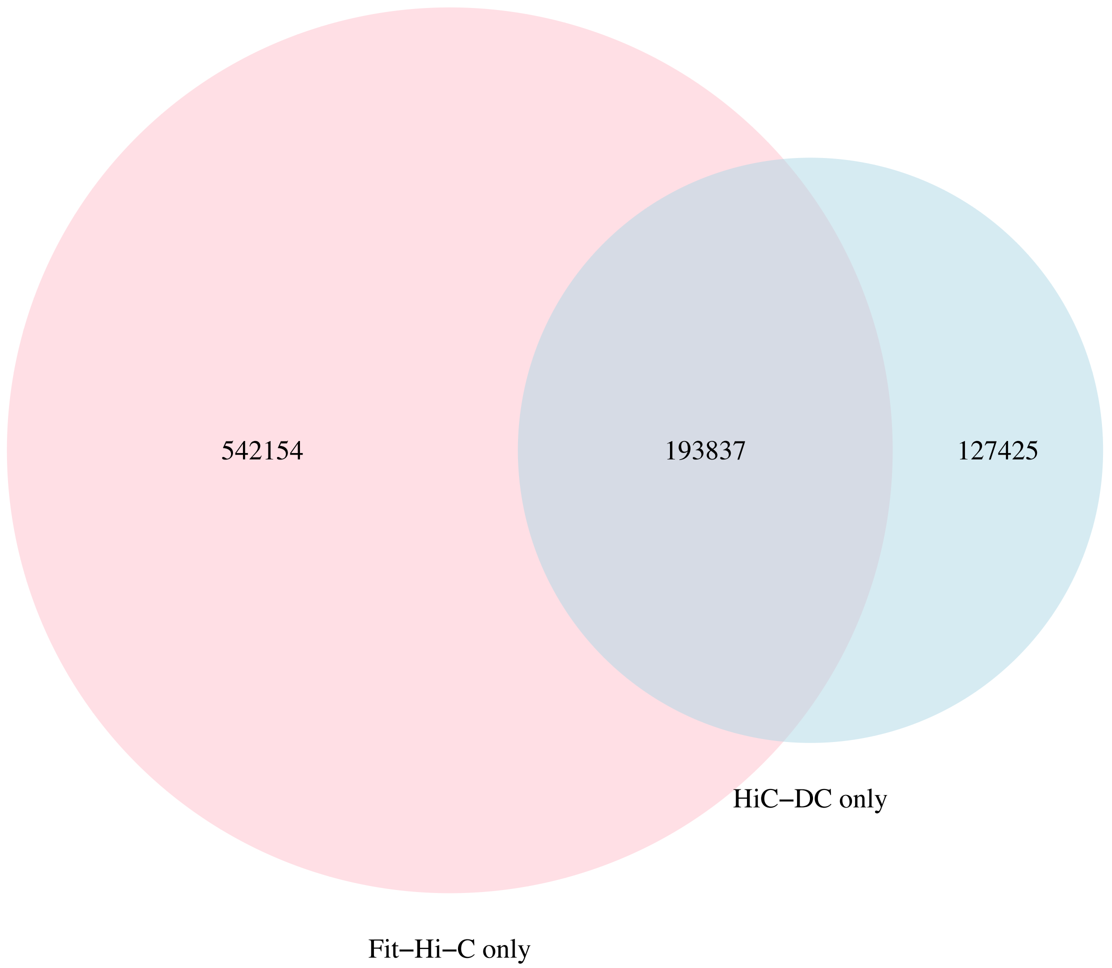

Supplementary Figure 6 – Overlap of significant contacts between Fit-Hi-C and HiC-DC
For 5kb fixed binned data from Rao et al.[1], we compared how many significant contacts are shared between Fit-Hi-C and HiC-DC genome-wide. Significant contacts were designated as those that had an FDR corrected *P* value less than 1% as assigned by each method.

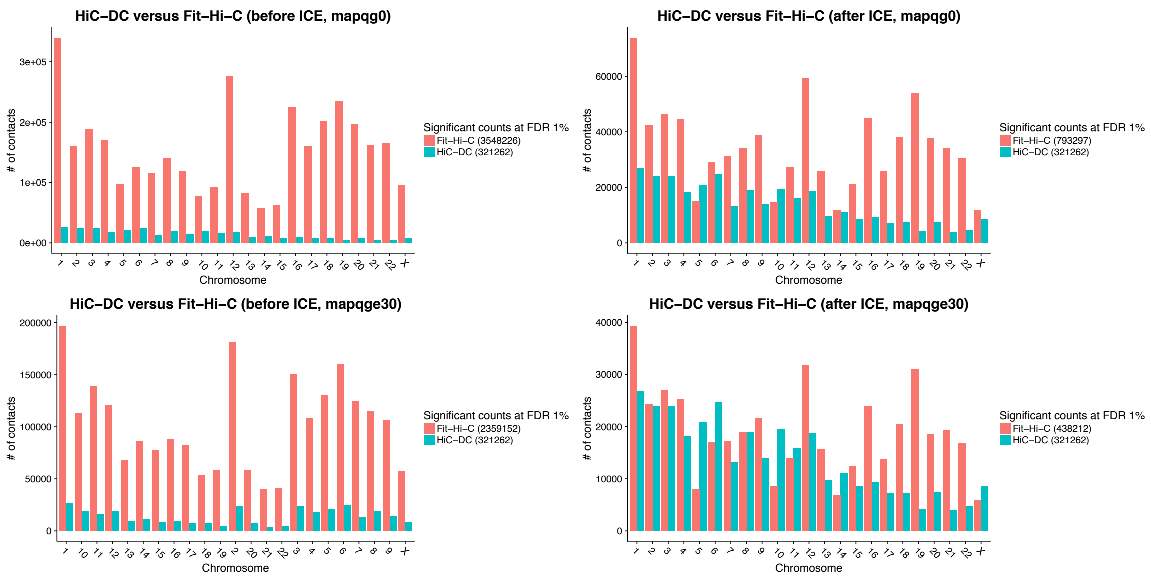

Supplementary Figure 7 – Effects of different MAPQ read quality thresholds and ICE normalization on statistical significance of Fit-Hi-C

For 5kb fixed binned data from Rao et al.[1], we computed how the number of significant contacts for Fit-Hi-C varied depending on the application of ICE, and the MAPQ read quality thresholds. Significant contacts are those which have an FDR adjusted *P* value less than 1%. The MAPQ thresholds correspond to a choice of read-count matrices provided by Rao et al.[1], and specify whether to include reads with a mapping quality greater than zero (MAPQG0), or only reads with mapping quality greater than or equal to 30 (MAPQGE30). This choice has a much smaller effect than the choice to use ICE normalized count matrices. For comparison, we include the number of significant contacts as determined by HiC-DC, which was run on MAPQG0 data.

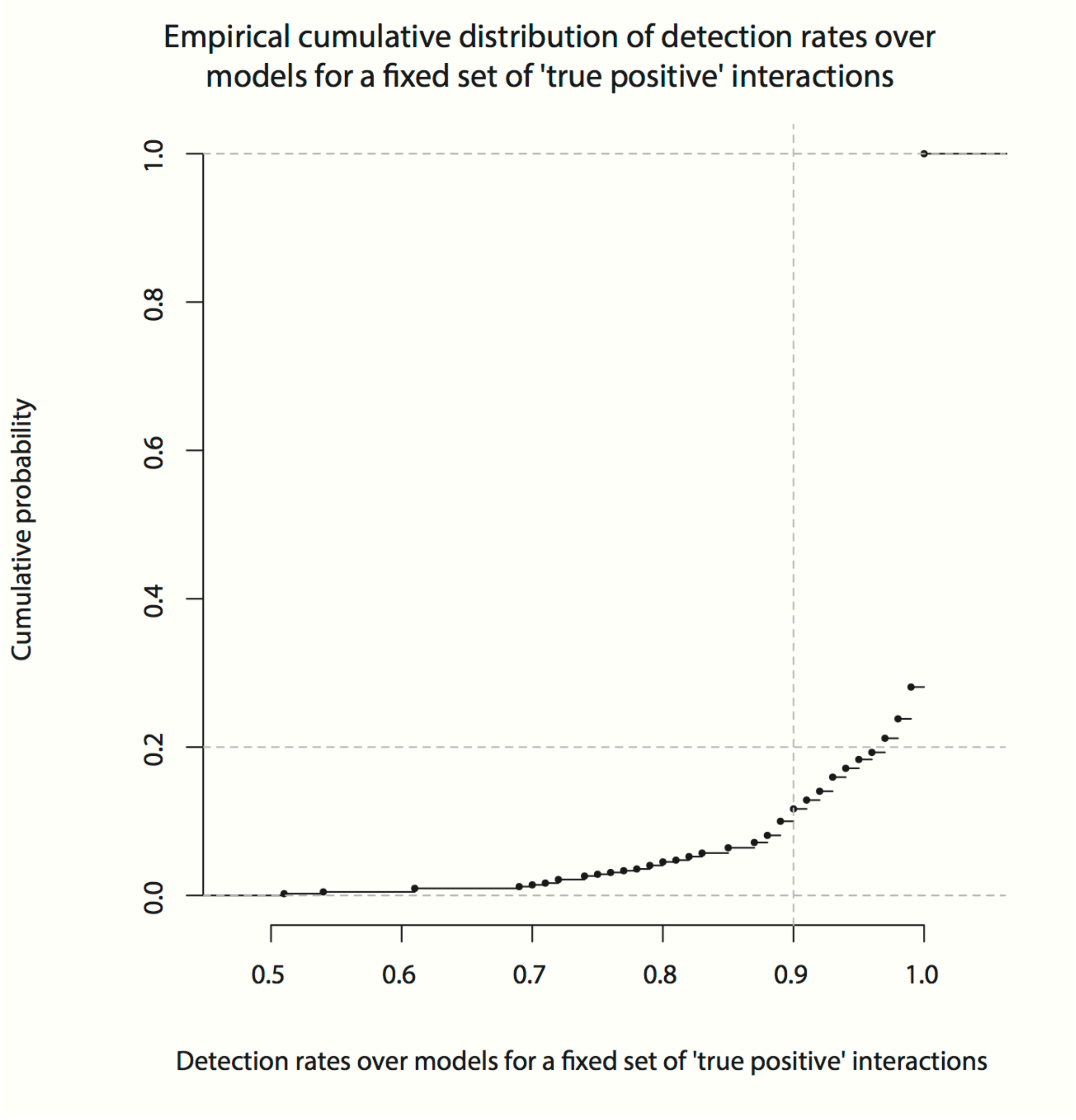

Supplementary Figure 8 – Reproducibility of statistically significant interactions detected by HiC-DC

HiC-DC was initially trained on a 1% subsample of interaction bins for GM12878, and we assigned significant interactions identified at 1% FDR threshold as our ground truth set. Then we randomly sampled 1% of interaction bins 100 times, trained a HiC-DC model on each sample, and computed how often each 'true positive' interaction was detected at 1% FDR by the 100 models. We plot the empirical cumulative distribution of detection rates over models for the set of 'true positives'. We performed this analysis on chromosome 21 at 5kb resolution for the Rao et al.[1] dataset.

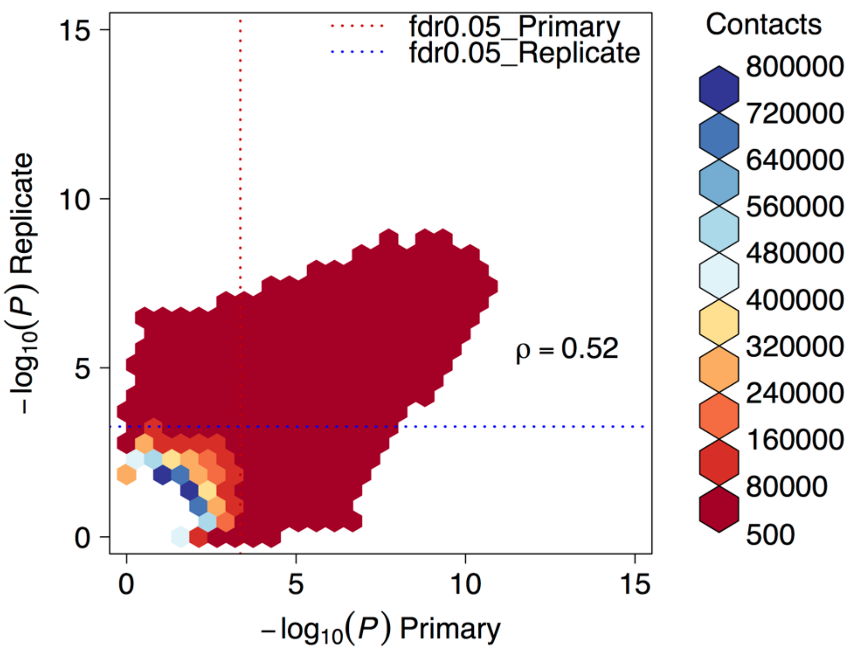

Supplementary Figure 9 – Scatterplot of –$\log_{10}$ *P*-values of all interactions as computed by HiC-DC on the Rao et al.[1] primary GM12878 replicate versus the secondary replicate
We used a 5kb fixed binning for both the primary and secondary GM12878 replicates from Rao et al.[1] for this analysis. The plot shows HiC-DC –$\log_{10}$ *P*-values for the primary replicate (x-axis) versus the secondary replicate (y-axis). Since the raw scatterplot is very dense because of the high number of contacts, it is represented here as contact densities in hexagonal bins. The red (primary) and blue (secondary) dotted lines show the 5% FDR thresholds.

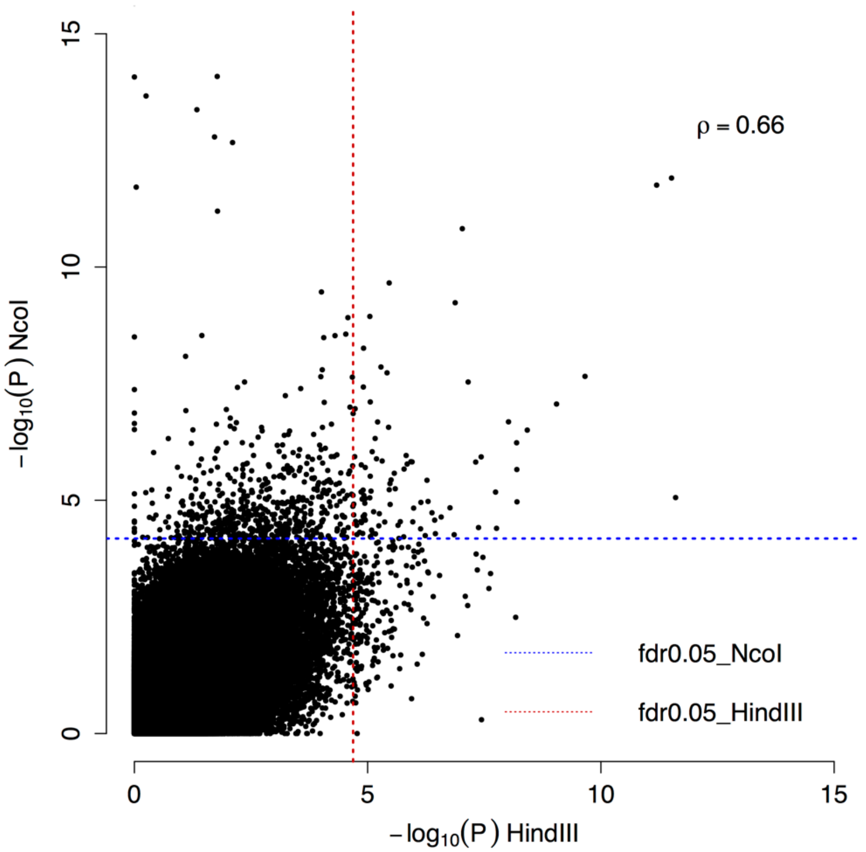

Supplementary Figure 10 – Scatterplot of –log$_{10}$ *P*-values of all interactions as computed by HiC-DC on Hi-C data using HindIII versus NcoI for restriction enzyme digestion at 50kb resolution

We used mouse ESC Hi-C data produced using either HindIII or NcoI by Dixon et al.[2] with uniform 50kb bins for this analysis. The plot shows HiC-DC –log$_{10}$ *P*-values for the HindIII data (x-axis) versus NcoI (y-axis). The red (HindIII) and blue (NcoI) dotted lines show the 5% FDR thresholds.

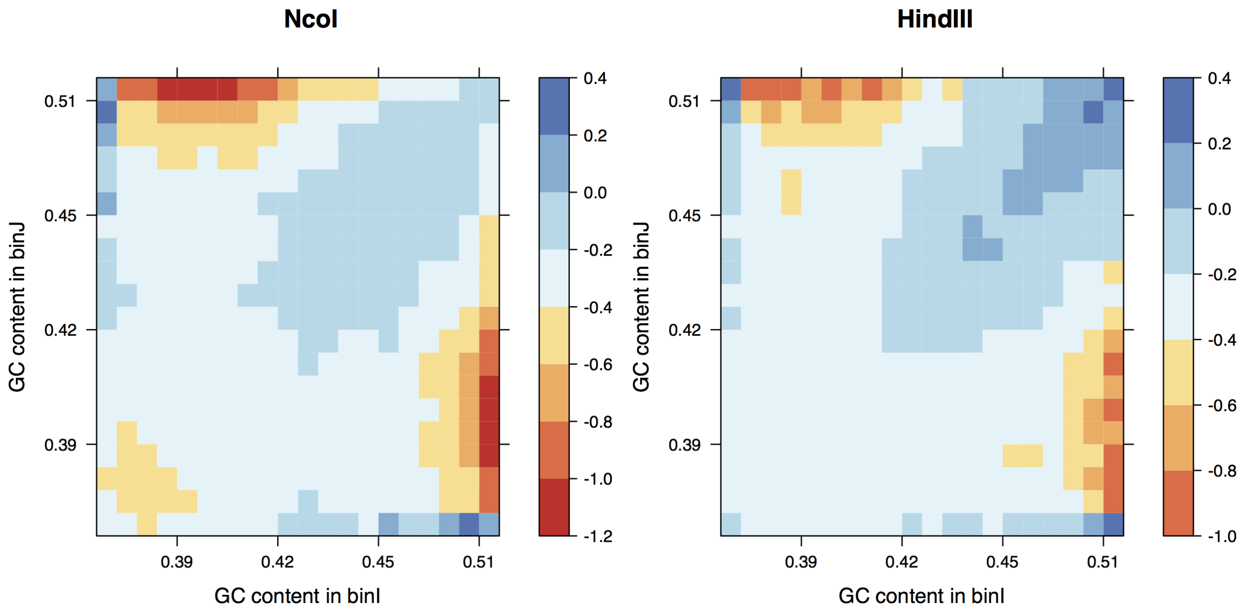

Supplementary Figure 11 – Hi-C contact enrichments stratified by GC content of the non-uniform bins using 10 RE sites on mouse ESC data from Dixon et al.[2]
We binned GC content into 20 equal sized bins. For each bin, we plot the $\log_2$ ratio of the mean observed count over mean expected count. The analysis shows that there are preferential Hi-C contact patterns among low regional GC content and high regional GC contact but that the bias is similar for the two different restriction enzymes.

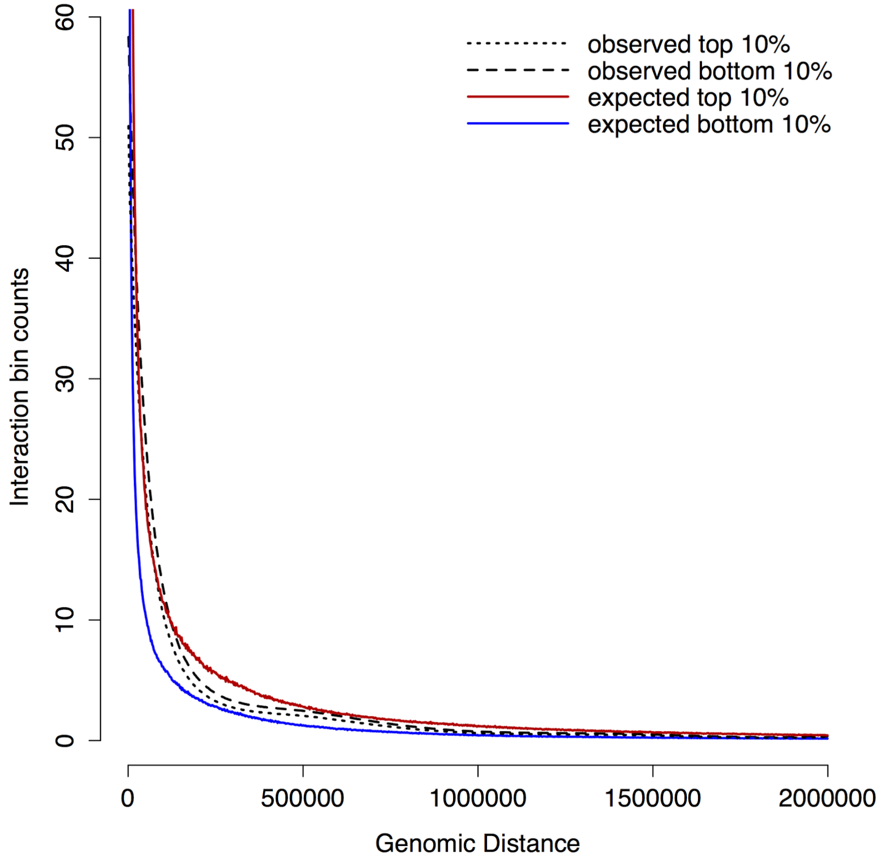

Supplementary Figure 12 – HiC-DC estimated means for bottom and top 10% quantiles for non-uniform (10 RE) bin sizes

We performed this analysis on the Rao et al.[1] GM12878 (primary replicate) Hi-C interaction data for chromosome 1. The expected and observed bin counts are plotted for the bottom and top 10% quantiles for non-uniform bin sizes. The dashed and dotted curves are observed bin counts for bottom and top 10% percentiles of bin sizes, respectively. The blue and red solid lines correspond to the estimated mean bin count for the bottom and top 10% percentiles according to the HiC-DC model.

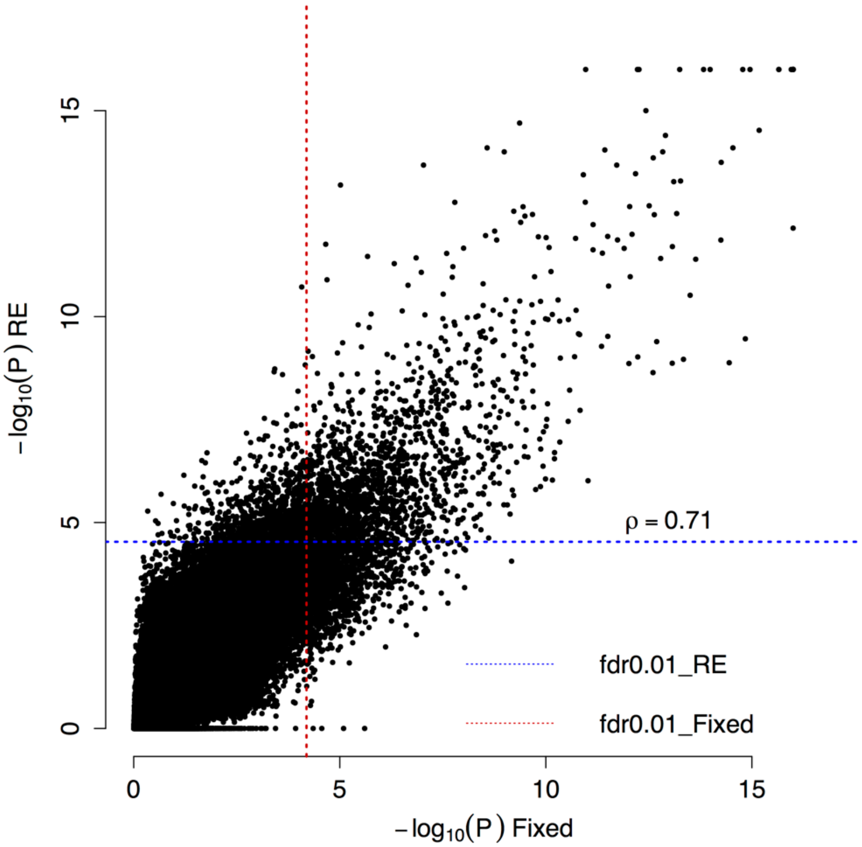

Supplementary Figure 13 – Scatterplot of –$\log_{10}$ *P*-values of significant interactions called by HiC-DC for non-uniform binning versus uniform binning

We used GM12878 (primary replicate) Hi-C data from Rao et al.[1] for this analysis. We considered bins with at least 85% overlap between uniform (5kb) and non-uniform (10 RE) bins and a minimum overlap length of 3kb for each pair of intervals. We plot the –$\log_{10}$ *P*-values for the uniform bin model (x-axis) versus the non-uniform bin model (y-axis) for interactions corresponding these overlapping bins. The red (uniform binning) and blue (non-uniform binning) dotted lines show the 1% FDR thresholds.

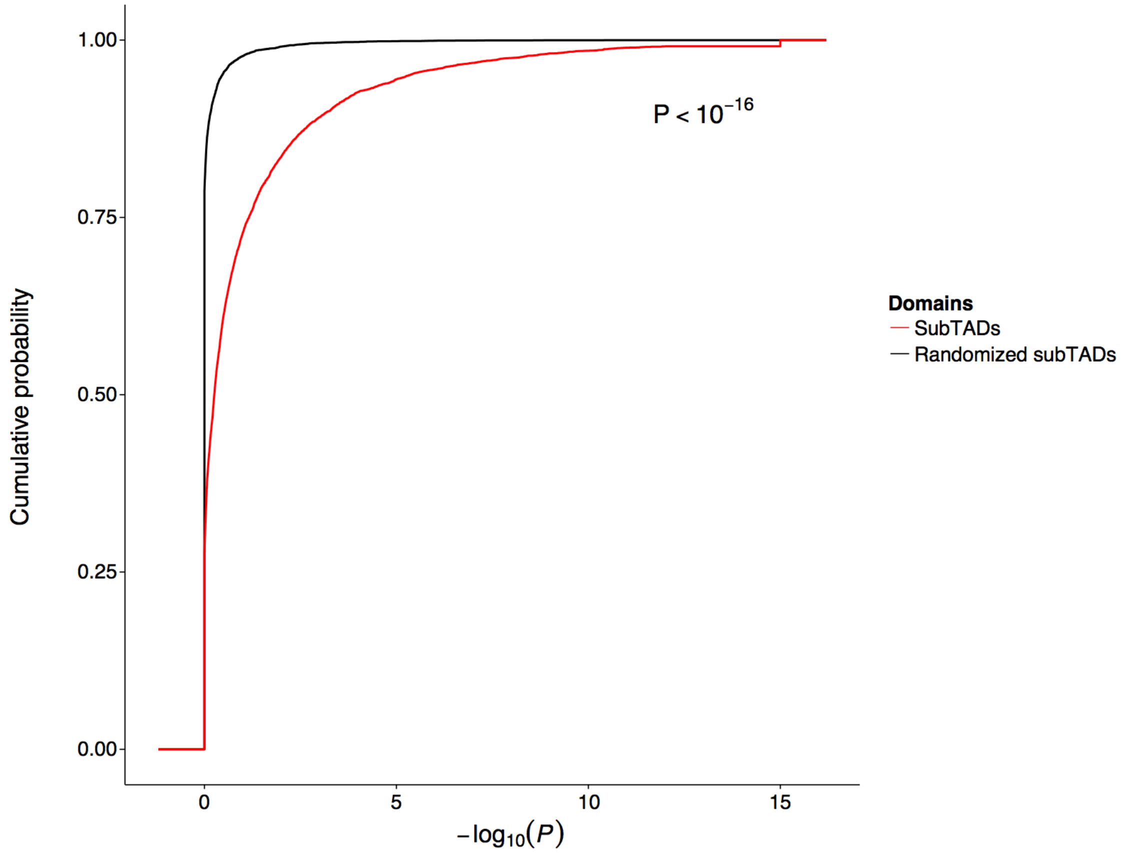

Supplementary Figure 14 – KS test of –log$_{10}$(*P*) distribution of true versus randomized sub-TAD corners

Genome-wide, 'corner' interactions of previously reported sub-TADs were strongly enriched for significant HiC-DC interactions. For this analysis, we identified all Hi-C pairs that connected the left and right corner of a sub-TAD reported by Rao et al.[1] and used the 10 RE fragment HiC-DC model. We created a randomized set of sub-TADs with the same length distribution as the reported sub-TADs. For each sub-TAD, we calculated a score that represents the maximum –log$_{10}$ *P* value of all Hi-C pairs connecting the corner of the sub-TAD. We tested for an enrichment of significant interactions using a Kolmogorov-Smirnov (KS) test.

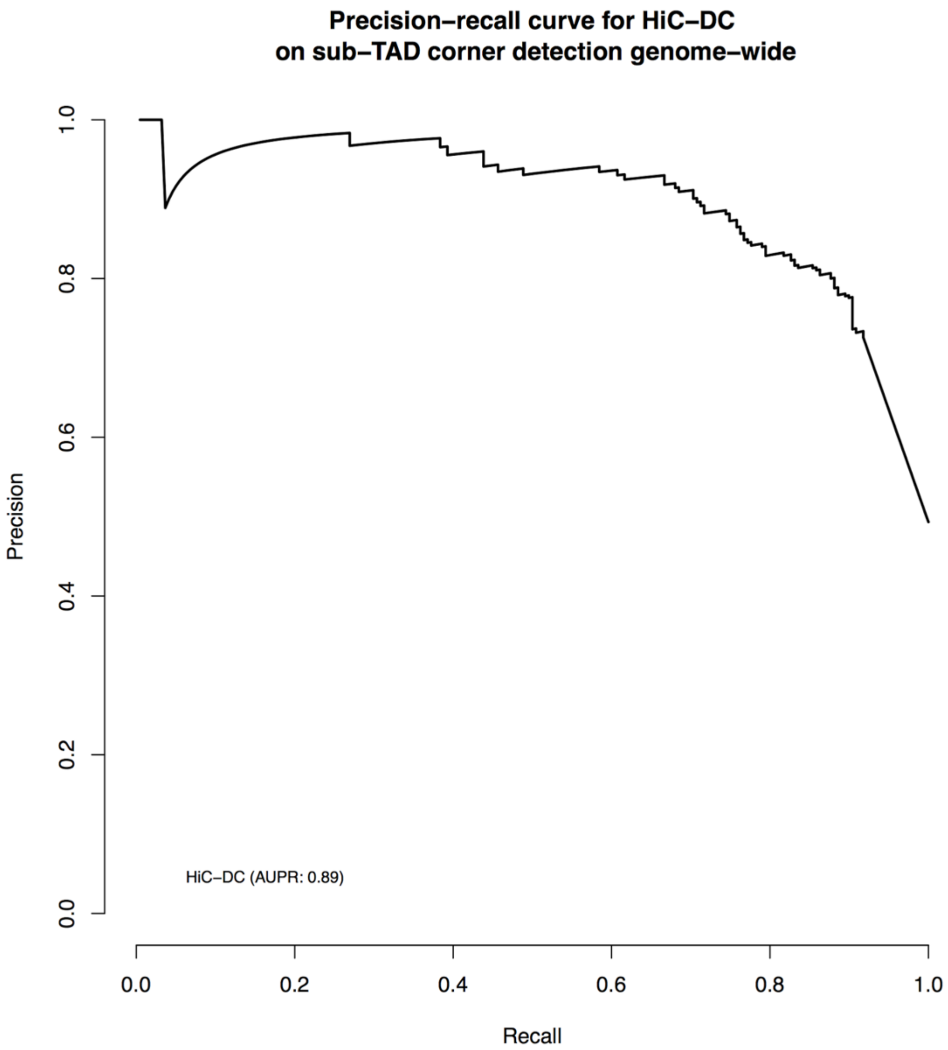

Supplementary Figure 15 – Precision-recall for HiC-DC on detection of sub-TADs
For this analysis, we identified all Hi-C interaction bins at 10 RE resolution that connected the left and right boundaries of a sub-TAD ("overlapping the sub-TAD corner") as reported by Rao et al.[1] using primary GM12878 Hi-C data. We created a randomized set of sub-TADs with the same length distribution as the reported sub-TADs. The sub-TADs reported by Rao et al.[1] and colleagues defined our ground-truth set; we labeled these sub-TADs a value of 1, and the randomized set of sub-TAD were labeled 0. For each sub-TAD, our predictions are defined by the maximum $-\log_{10} P$ value of all Hi-C interaction bins overlapping the corner of the sub-TAD, and we plotted corresponding precision-recall curve.

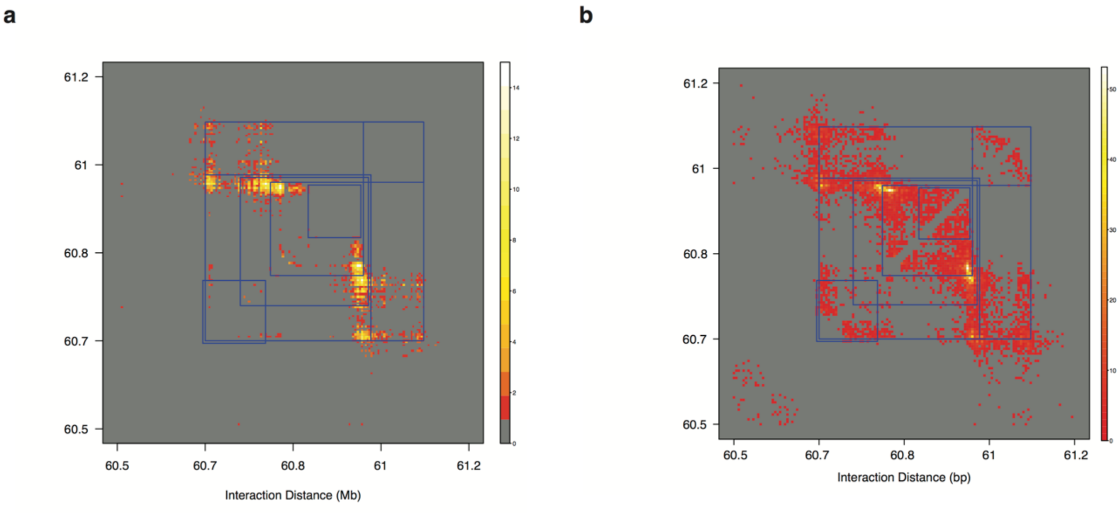

Supplementary Figure 16 – *BCL2* locus HiC-DC *P* values versus Fit-Hi-C *P* values

(a) Hi-C interaction matrix of $-\log_{10} P$ values for each contact in a ~700kb region of chromosome 18 containing the gene *BCL2*, as calculated by HiC-DC. **(b)** Hi-C interaction matrix in the same region, $-\log_{10} P$ values calculated by Fit-Hi-C. Previously reported sub-TADs by Rao et al.[1] are drawn in blue boxes.

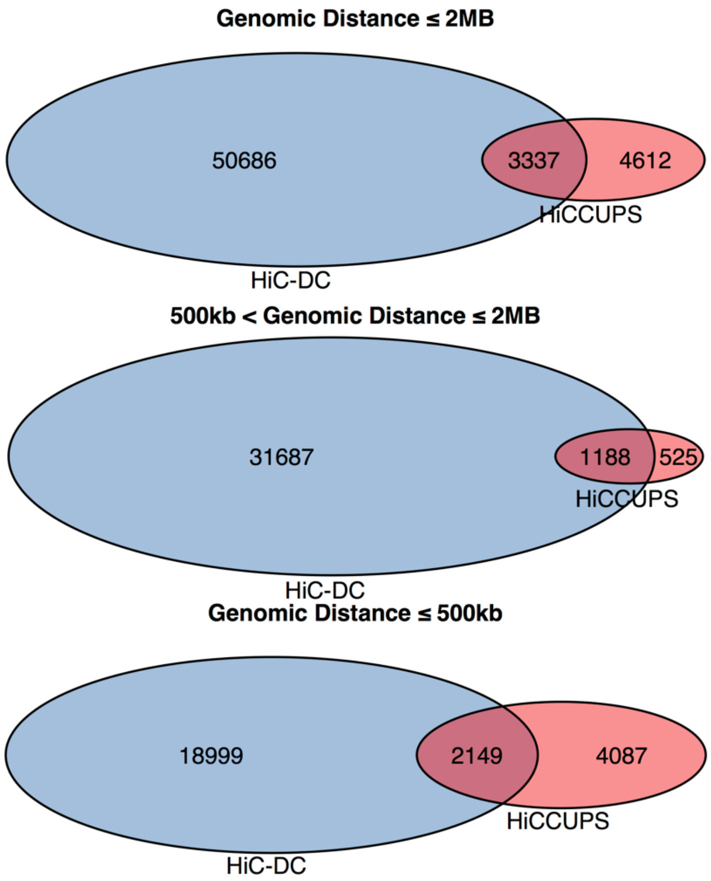

Supplementary Figure 17 – Comparison of significant interactions called by HiC-DC and HiCCUPS

We compared interactions reported by HiCCUPS to our significant HiC-DC interactions identified at 5% FDR threshold on the Rao et al.[1] GM12878 data binned at 10kb resolution to better match the resolution of HiCCUPS. We repeated this comparison for detected significant interactions (excluding diagonal interaction bins) within 2Mb, within 500kb, and in between these two distances. HiCCUPS calls are concentrated at smaller genomic distances (<500kb) while the majority of significant HiC-DC interactions occur at larger genomic distances (>500kb). Of the 4612 interactions not detected as significant by HiC-DC but reported by HiCCUPs, 47% (2179) have at least two constituent 5kb bins (out of four) called as significant by HiC-DC when run at 5kb resolution.

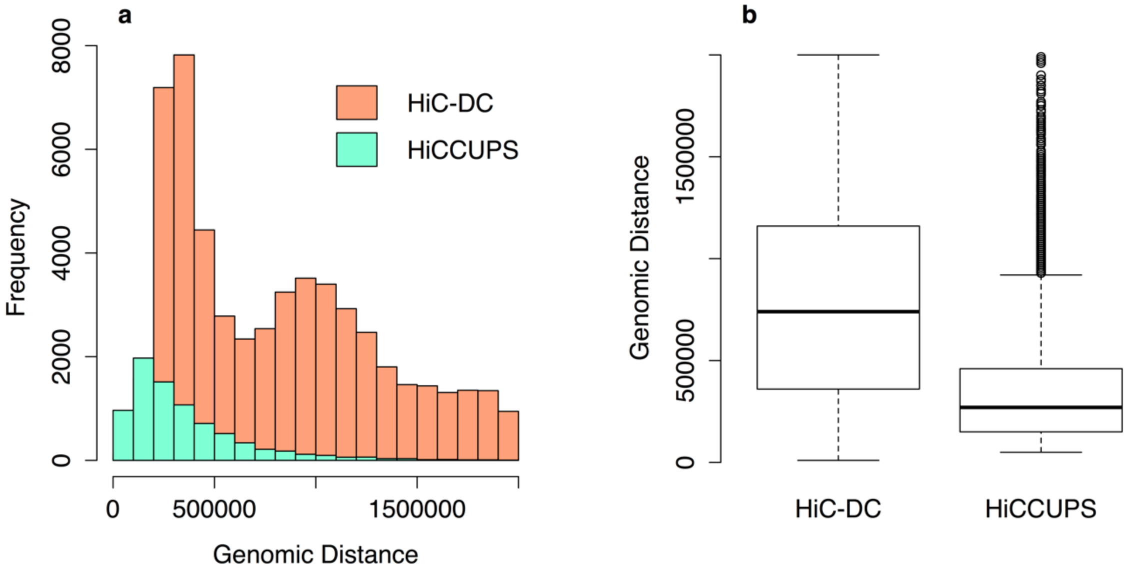

Supplementary Figure 18 – Distribution of significant interaction calls by HiC-DC and HiCCUPS over genomic distance

The left panel shows the distribution of significant interactions reported by HiCCUPS and our significant interactions (excluding diagonal interaction bins) identified at 5% FDR threshold Rao et al.[1] GM12878 data binned at 10 kb resolution. The panel on the right illustrates the genomic distance distributions of significant interactions as boxplots. HiC-DC and HiCCUPS cover interactions in complementary genomic distance ranges. The median genomic distance of detected significant interactions by HiC-DC (~740kb) corresponds to the 90th percentile of that of HiCCUPS.

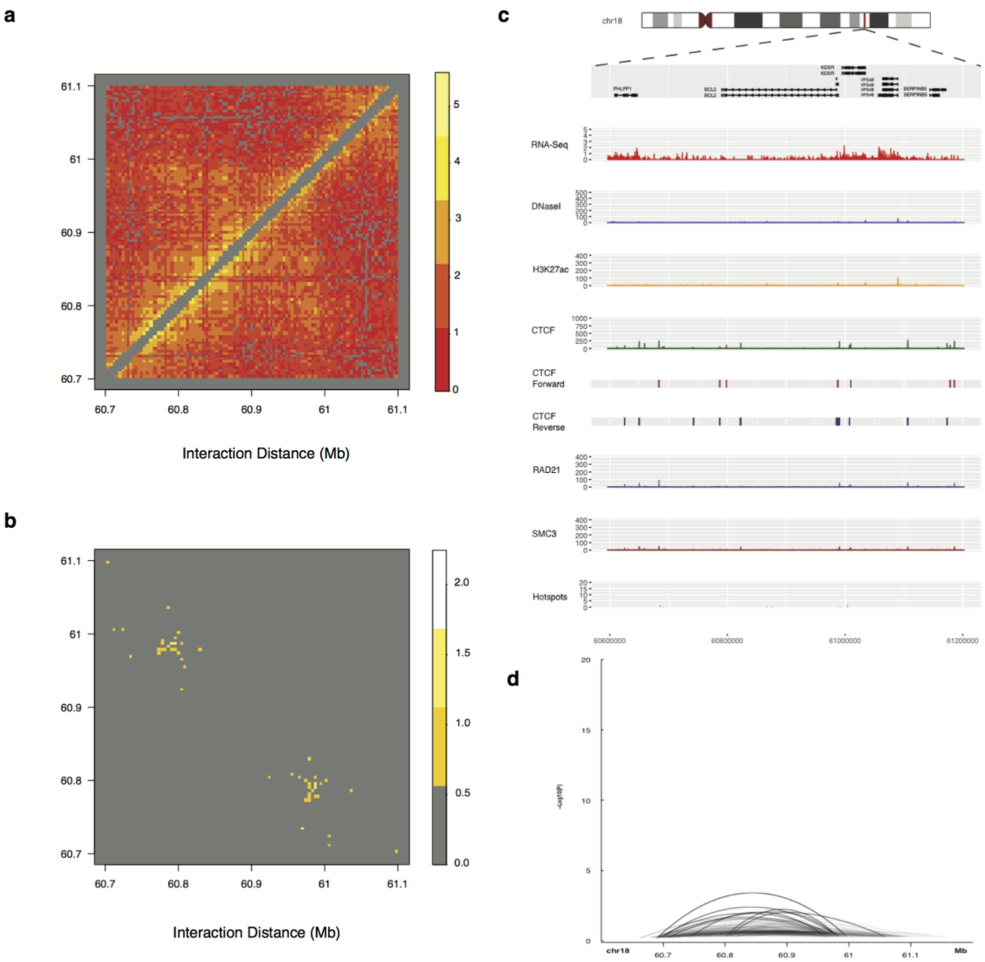

Supplementary Figure 19 – Fine mapping of chromatin interactions involving BCL2 locus in K562

ENCODE signal tracks representing RNA-Seq, DNaseI, H3K27ac, RAD21, CTCF, and SMC3 peaks in K562. We summarized maximum $-\log_{10} P$ values within a 700 kb region of the *BCL2* locus as 1D signal track, which we labeled as "HiC hotspots" (see main text). In addition, we also show the forward (red) and reversed (blue) orientations of the CTCF motif. The sashimi plot represents significant interactions identified by HiC-DC, and the height of the arc denotes the level of significance ($-\log_{10} P$ value).

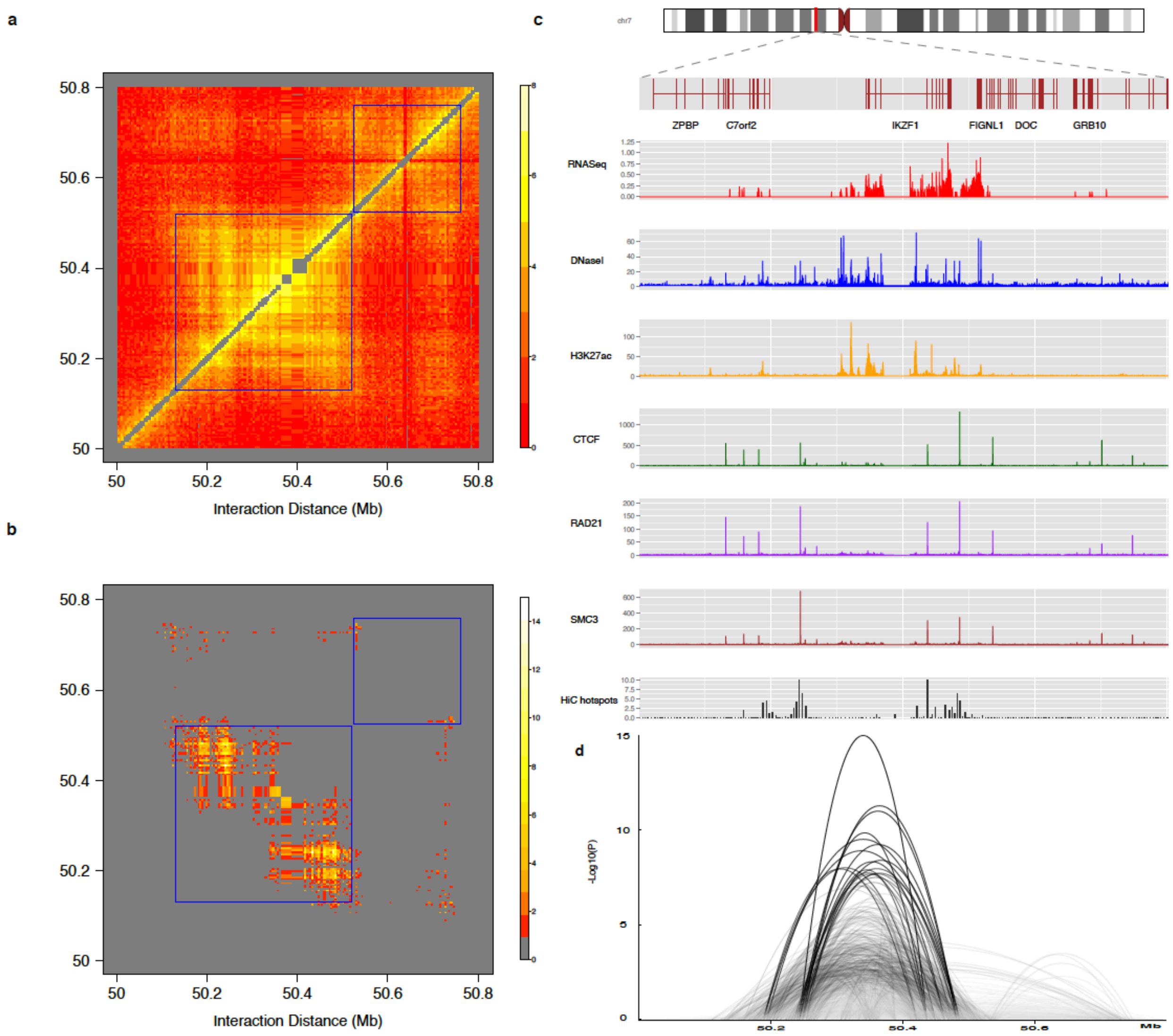

Supplementary Figure 20 – *IKZF*1 is flanked by upstream and 3' end / downstream Hi-C hotspots, all co-occupied by CTCF and cohesin

(a) The raw Hi-C interaction count matrix for a ~800kb region encompassing the *IKZF* gene on chromosome 7, with previously reported sub-TADs by Rao et al.[1] drawn as blue boxes. **(b)** The $-\log_{10} P$ values for contacts as reported by HiC-DC. **(c)** ENCODE derived signal tracks spanning the same region as panels (a) and (b), displaying RNA-seq, DNaseI-seq, H3K27ac-seq, as well as ChIP-seq data for CTCF, RAD21 and SMC3 respectively. The last track represents the 1D Hi-C hotspot track of the most significant interactions (see main text). **(d)** An arc plot of significant Hi-C interactions reported by HiC-DC. Each arc represents a significant interaction, and the height of arc indicates the level of statistical significance ($-\log_{10} P$ value) assigned by HiC-DC.

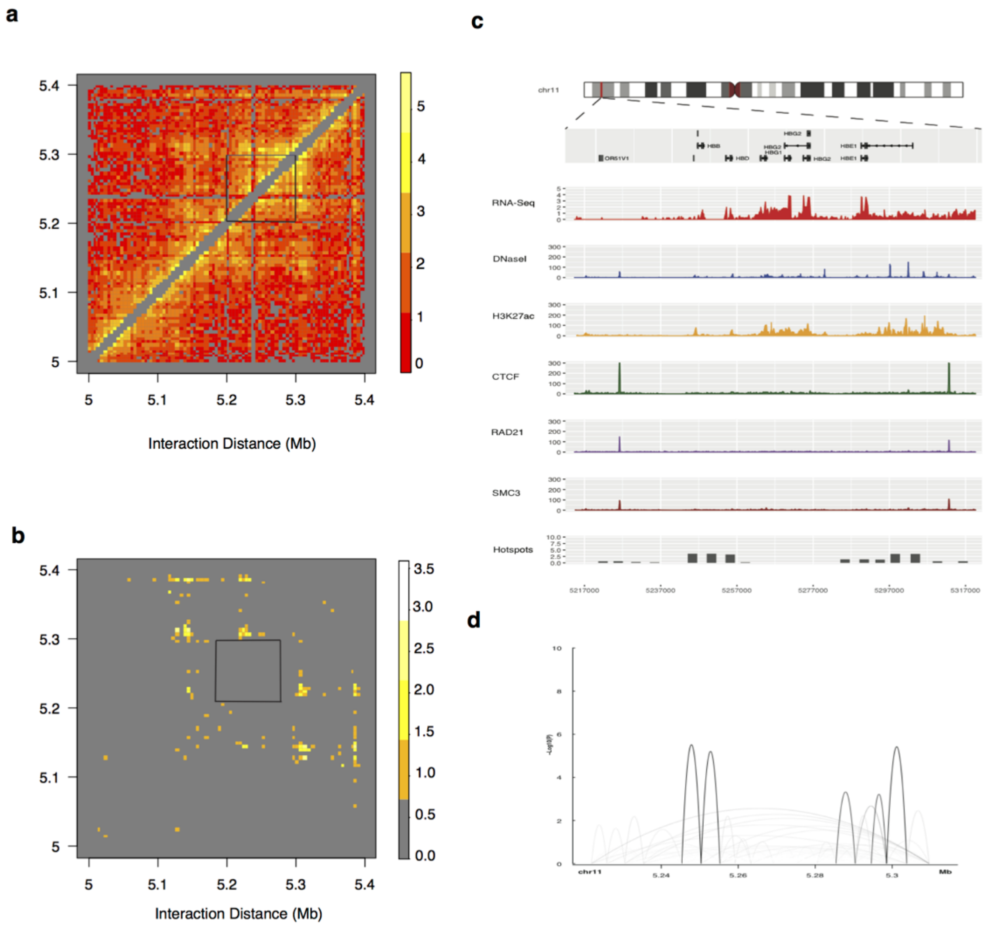

Supplementary Figure 21 – Interactions associated with locus control region of beta-globin gene cluster in K562

Similar to plots for *BCL2* and *IKZF1*, we show derived epigenetic and genome signal tracks from ENCODE together with Hi-C hotspots within a 100 kb region containing the beta-globin locus.

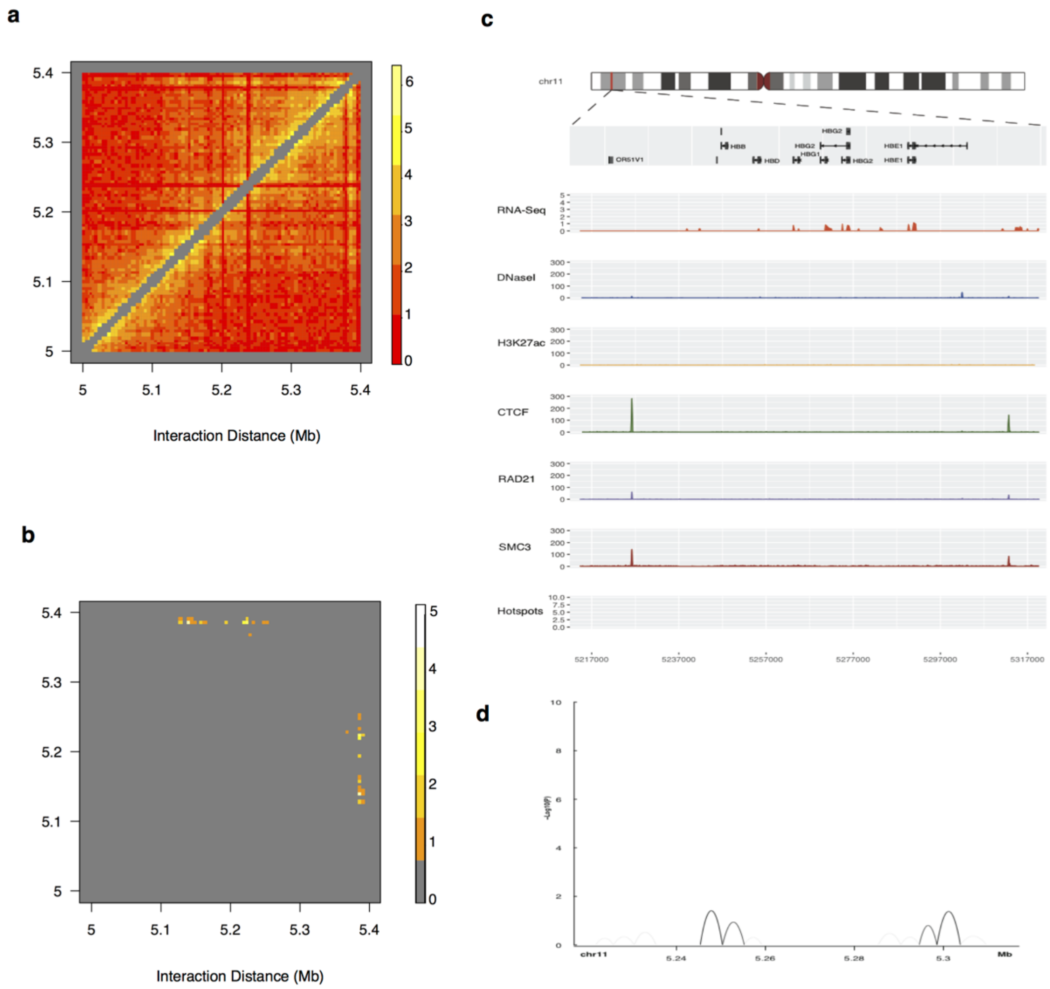

Supplementary Figure 22 – DNA interactions involving the beta-globin locus in GM12878
Similar to **Supplementary Figure 21**, we show the beta-globin locus in lymphoblastoid cells. Significant interactions are summarized as a 1D signal track, which is shown together with epigenetic and genomic signal tracks from ENCODE GM12878 data.

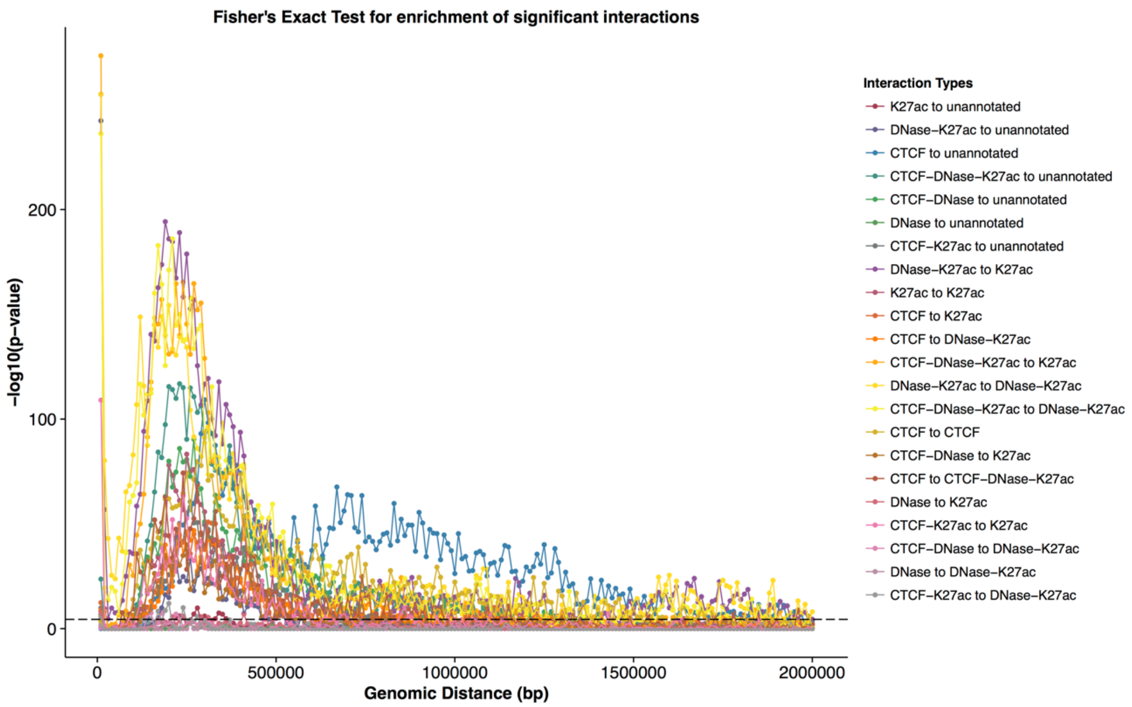

Supplementary Figure 23 – Enrichment *P* values for epigenetic signals

Enrichments of significant interactions (FDR < 1%) for the Rao et al.[1] data as determined by HiC-DC, annotated by epigenetic signals, as a function of genomic distance. For each 10kb band, enrichment of interactions with each specific annotation was computed relative to the background prevalence of this annotation (Fisher's exact test, see also **Methods**). DNase related contacts dominate over short distances (0 – 500kb), while CTCF related contacts are relatively more prevalent over medium distances (500kb – 1.5Mb).

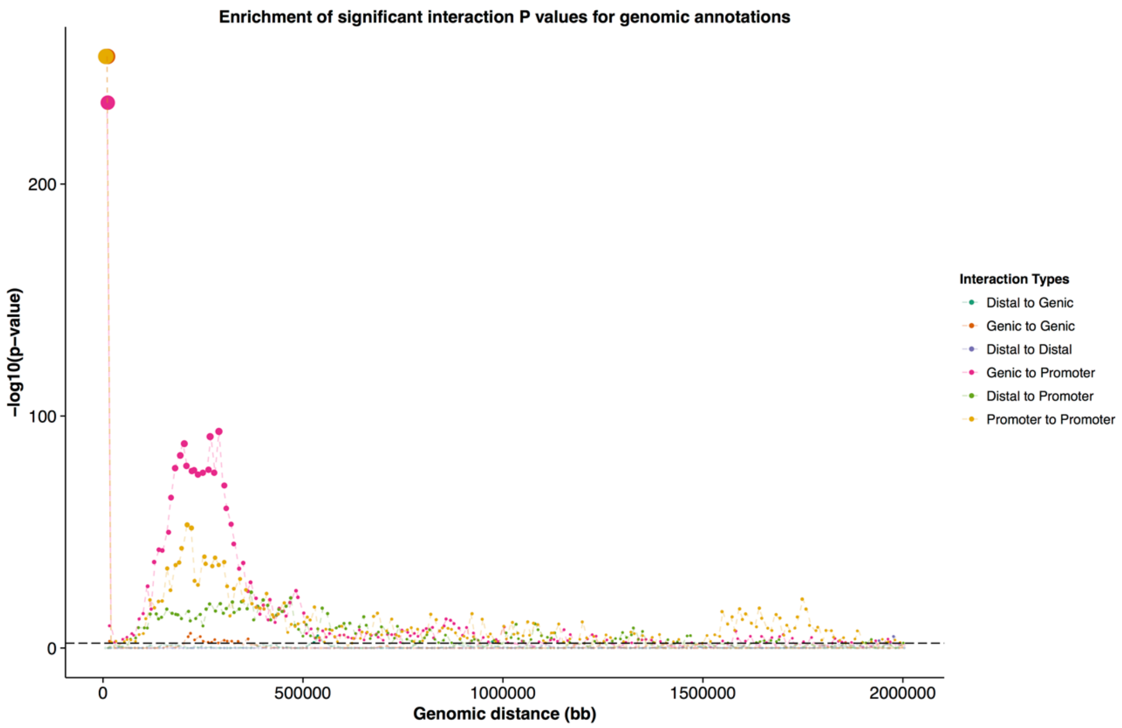

Supplementary Figure 24 – Enrichment *P* values for genomic annotations

Enrichments of significant interactions (FDR < 1%) for the Rao et al.[1] data as determined by HiC-DC, annotated by genomic location (promoter, gene body, or distal intergenic) as a function of genomic distance. Enrichment of interactions for each 10kb band was computed as in **Supplementary Figure 23**. Genic to promoter contacts dominate over short distances (0 – 500kb), and promoter to promoter contacts show a strong enrichment at long range interactions (1.5Mb – 2Mb).

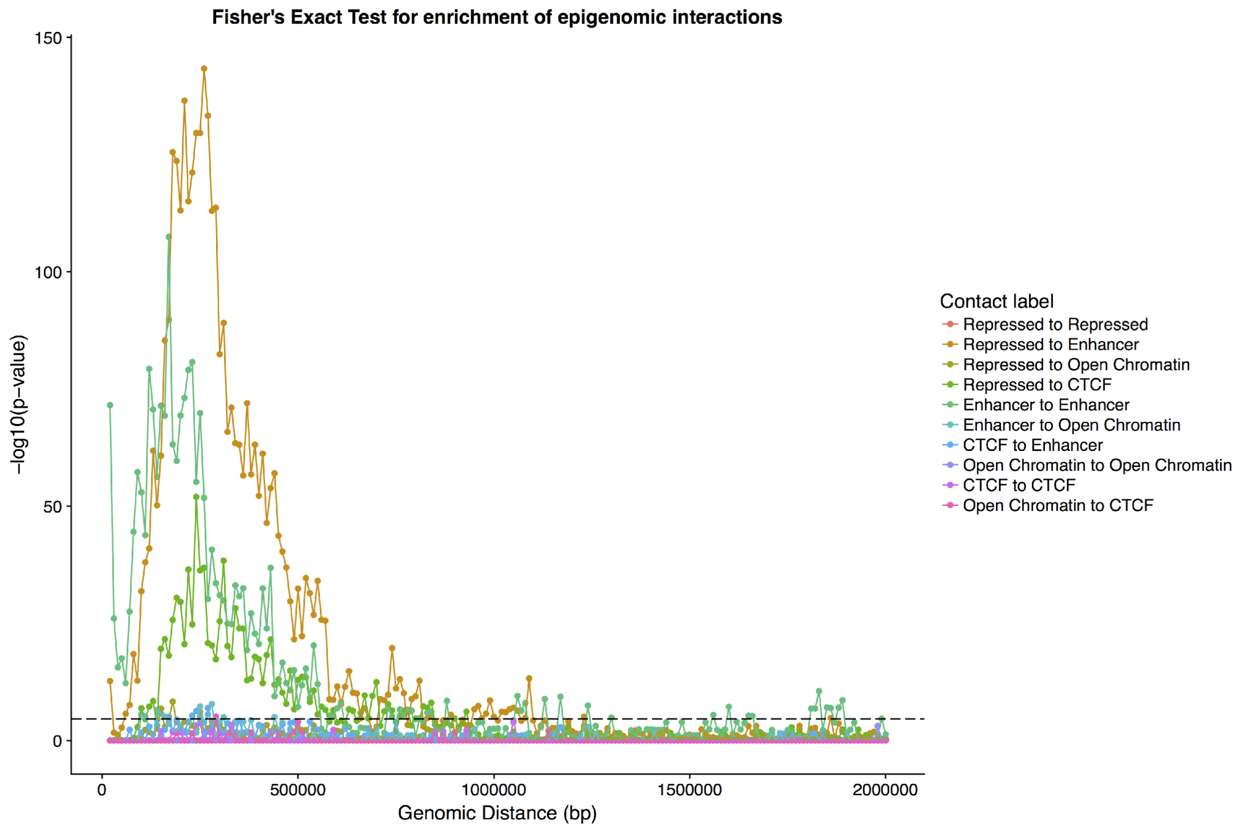

Supplementary Figure 25 – Fisher's exact test for enrichment of epigenomic interactions
We downloaded the combined ChromHMM and Segway annotations for GM12878 from UCSC. We then re-annotated genomic intervals used to define interaction bins by choosing the annotation of the ChromHMM + Segway region which had maximal overlap with each interval, restricting to annotations related to epigenomic states. After applying these new annotations, we recomputed the epigenomic enrichment analysis as previously described in the **Methods**.

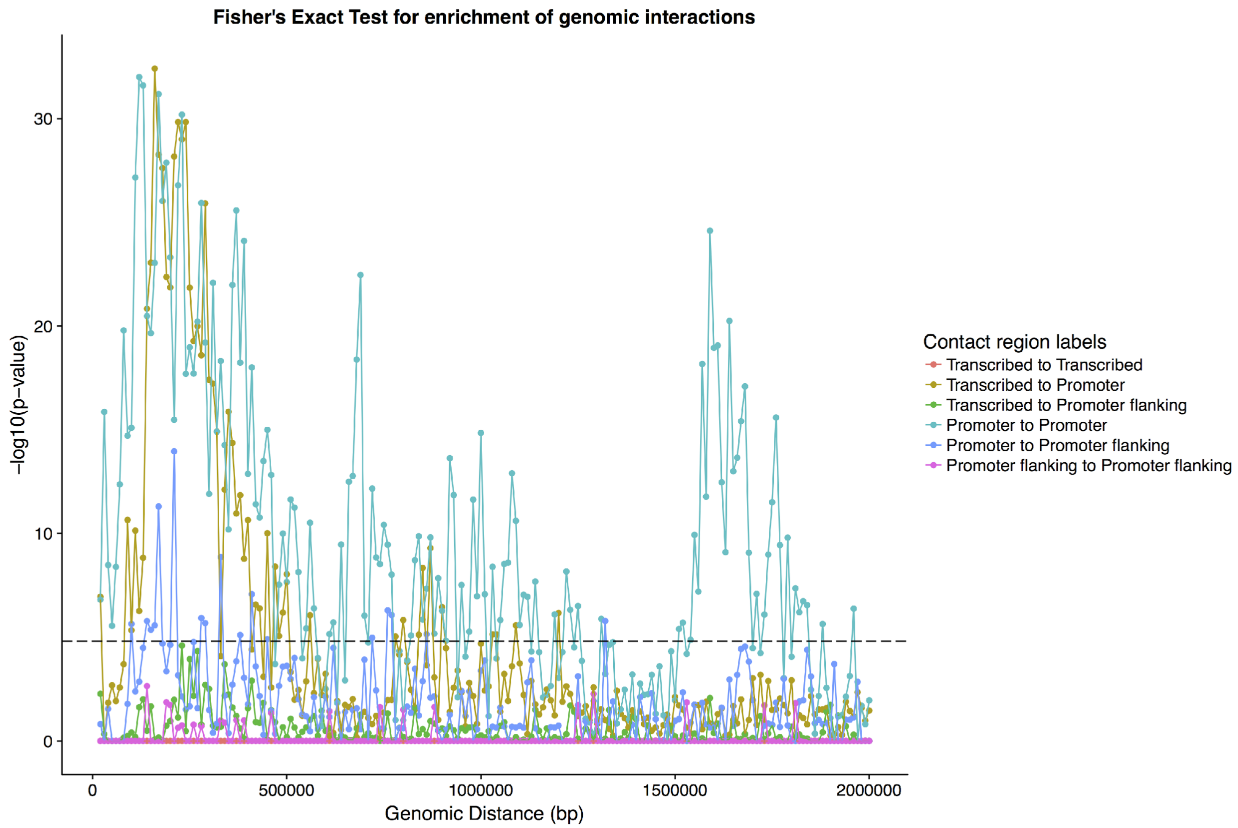

Supplementary Figure 26 – Fisher's exact test for enrichment of genomic interactions
We downloaded the combined ChromHMM and Segway annotations for GM12878 from the UCSC Genome Browser website. We then re-annotated genomic intervals used to define interaction bins by choosing the annotation of the ChromHMM + Segway regions which had maximal overlap with each interval, restricting to states related to genomic annotations. After applying these new annotations, we recomputed the genomic enrichment analysis as previously described in the **Methods**.

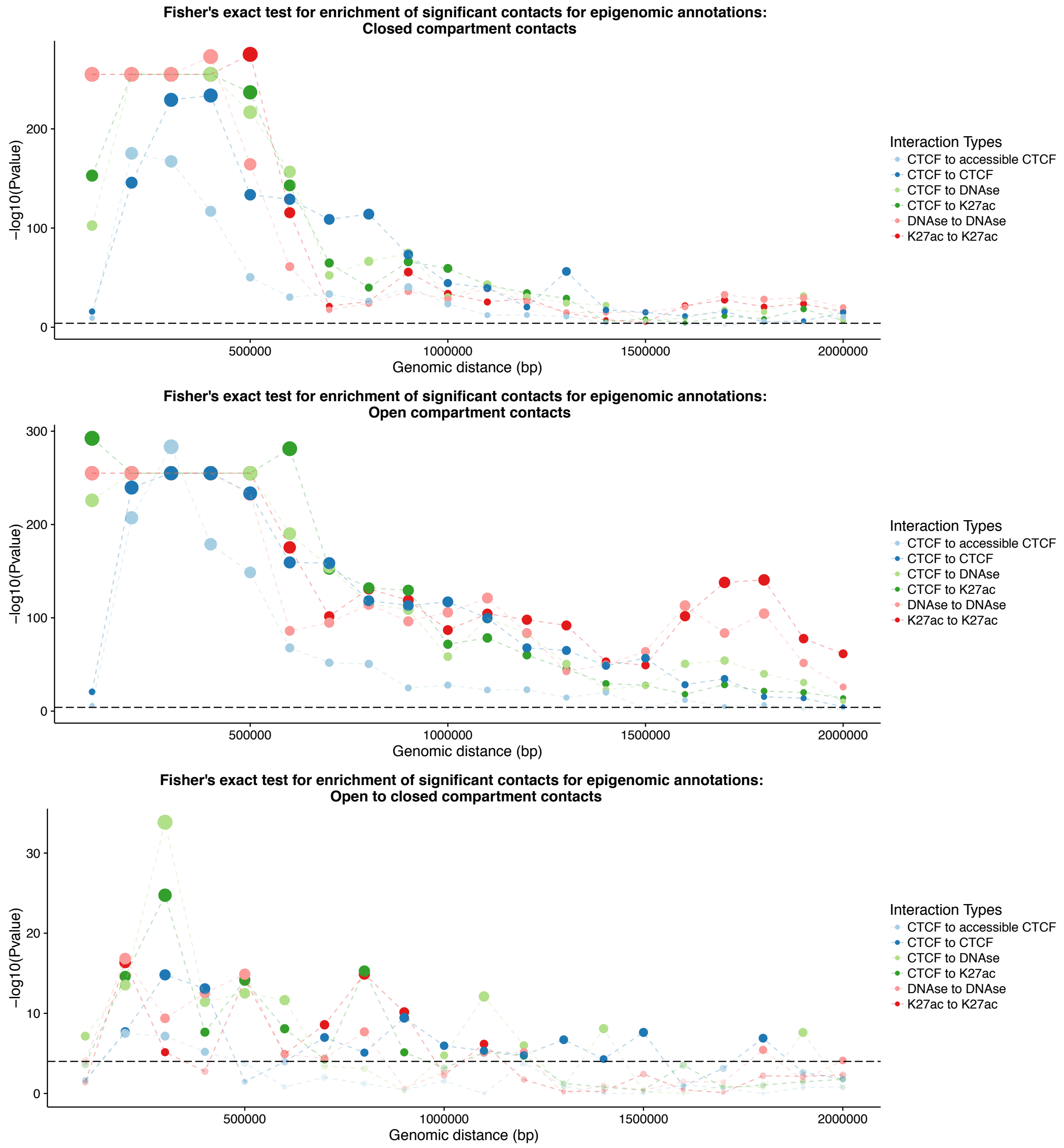

Supplemental Figure 27 – DNA looping events annotated by epigenetic marks demarcated by open and closed genomic compartments

Enrichments of significant interactions (FDR < 1%) for the Rao et al.[1] data as determined by HiC-DC, stratified by compartment and annotated by epigenomic signal as a function of genomic distance. Each intra-chromosomal contact matrix was binned at 100kb resolution, and compartment labels A (open) or B (closed) for the meta-bins were assigned (see **Methods**). Contacts were partitioned according to bin label into open to open, closed to closed, and open to closed sets. Each set of enrichment of interactions for each 10kb band was calculated as in **Supplementary Figure 23**. Open to open contacts show a marked enrichment for DNase hypersensitive sites as well as H3K27ac marks relative to either closed or open to closed contacts.

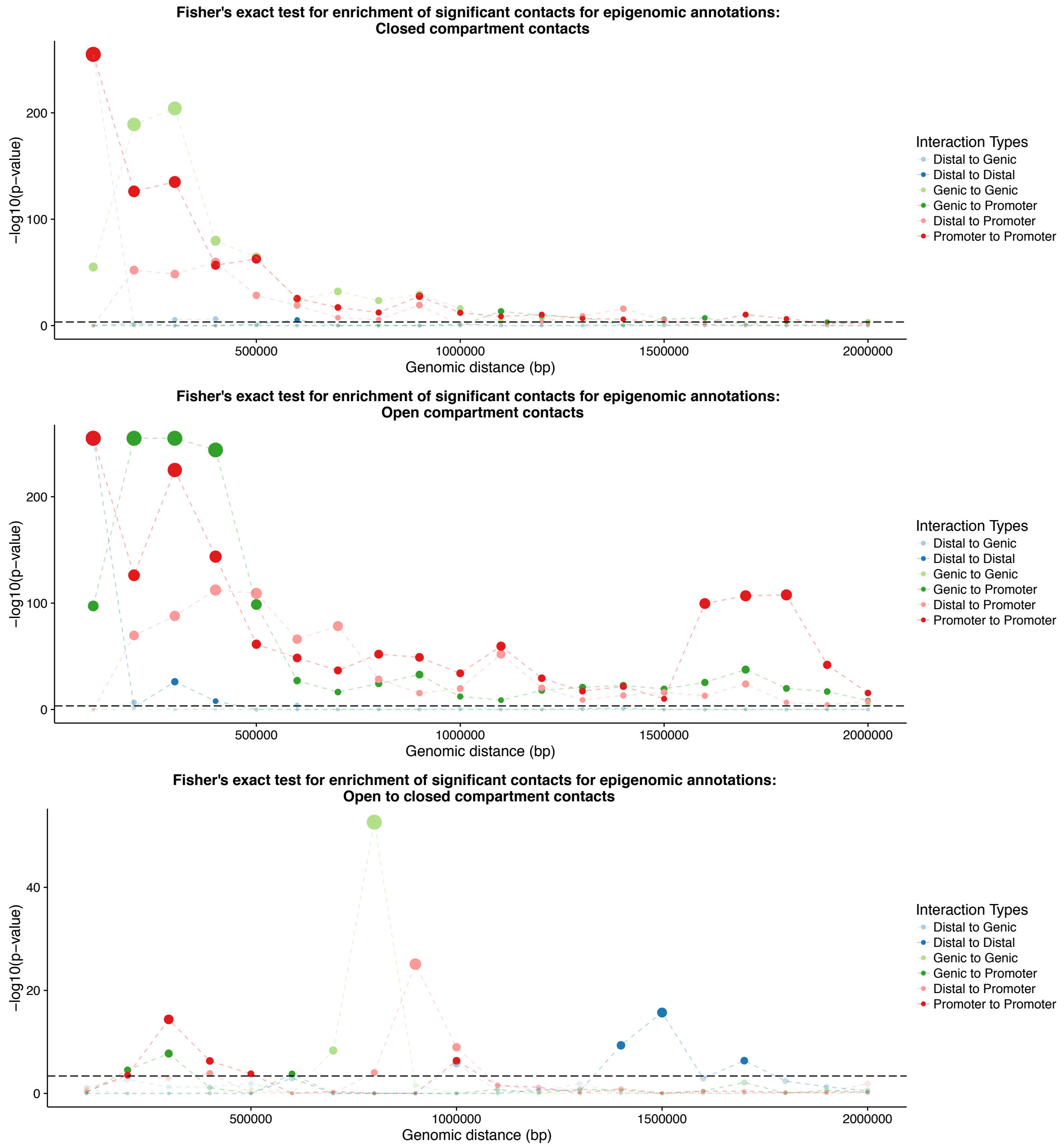

Supplemental Figure 28 – Chromatin interactions annotated by genomic location segregate into A/B compartments

Enrichments of significant interactions (FDR < 1%) for the Rao et al.[1] data as determined by HiC-DC, stratified by compartment and annotated by genomic location (promoter, gene body, or distal intergenic) as a function of genomic distance. Each intra-chromosomal contact matrix was binned at 100kb resolution, and compartment labels A (open) or B (closed) for the meta-bins were assigned (see **Methods**). Contacts were partitioned according to bin label into open to open, closed to closed, and open to closed sets. Each set of enrichment of interactions for each 10kb band was calculated as in (**Supplementary Figure 23**). Open to open contacts show a marked enrichment for genic to promoter over short distances (< 500kb), and for promoter-to-promoter contacts over long distances (>1.5Mb).

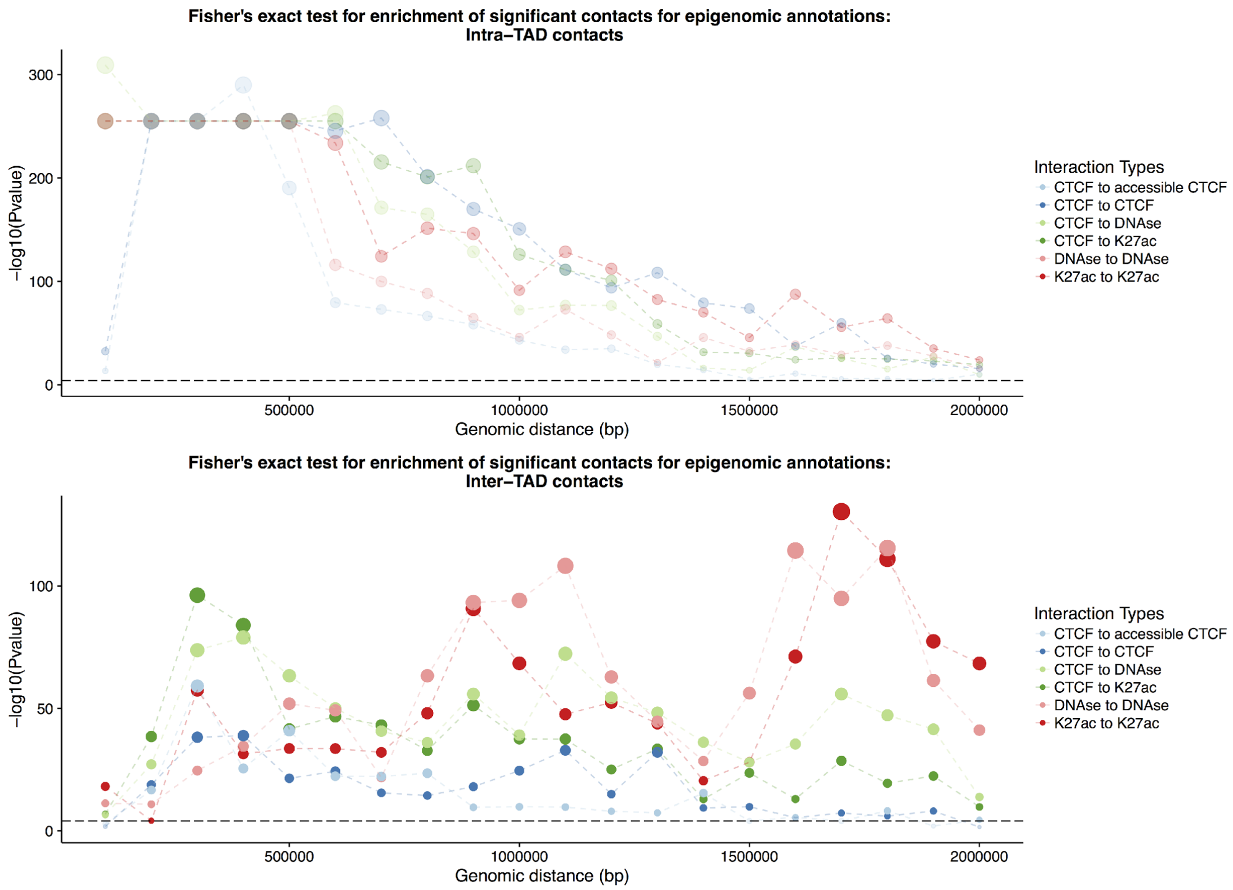

Supplementary Figure 29 – Fisher's exact test for enrichment of epigenomic annotations stratified by TAD

TAD coordinates data were taken from Dixon et al.[2], converted to hg19 via lift over, and used to partition HiC-DC contacts into those which spanned TAD boundaries (inter-TAD) and those which lay within TADs (intra-TAD). After partitioning, we re-ran the enrichment analysis as described in the **Methods**.

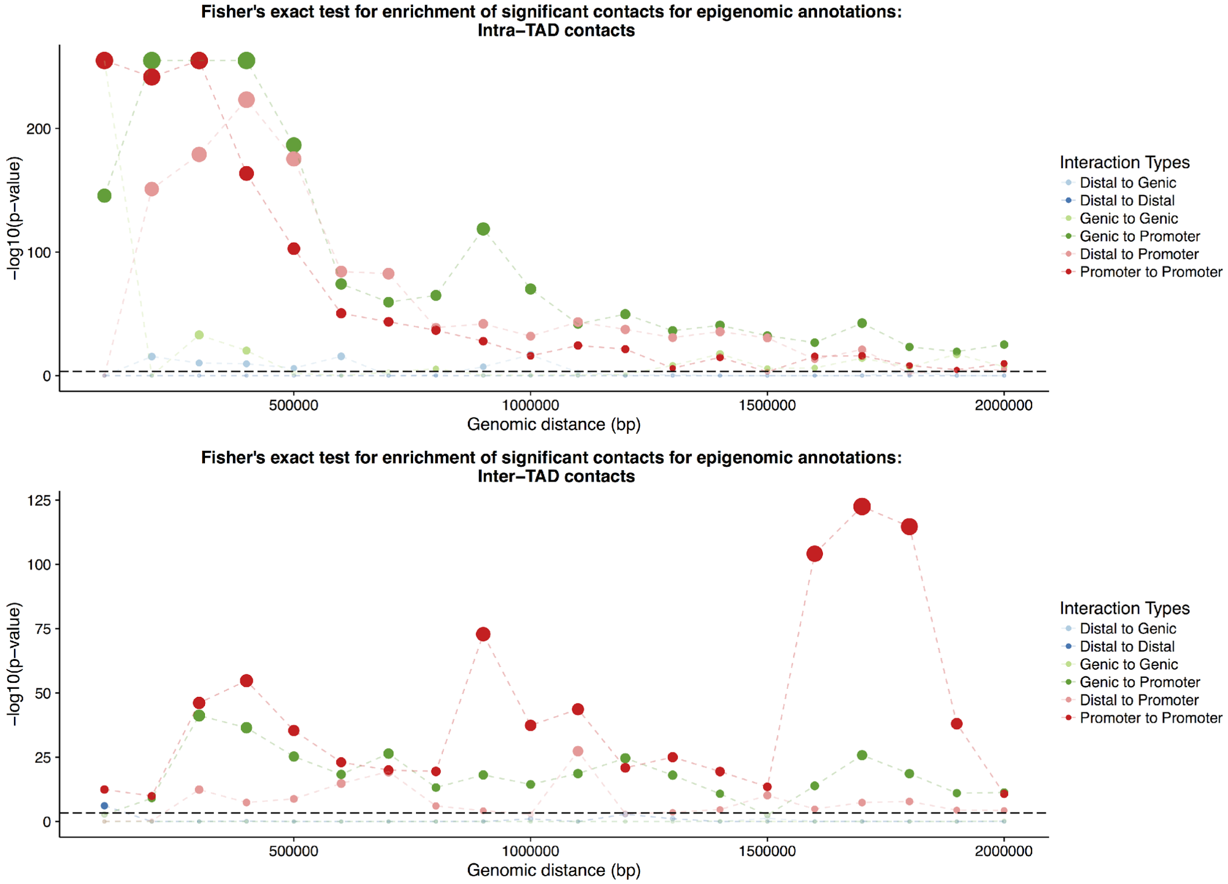

Supplementary Figure 30 – Fisher's exact test for enrichment of genomic annotations stratified by TAD

TAD coordinates data were taken from Dixon et al.[2], converted to hg19 via liftover, and used to partition HiC-DC contacts into those that spanned TAD boundaries (inter-TAD) and those that lay within TADs (intra-TAD). After partitioning, we re-ran the enrichment analysis as described in the **Methods**.

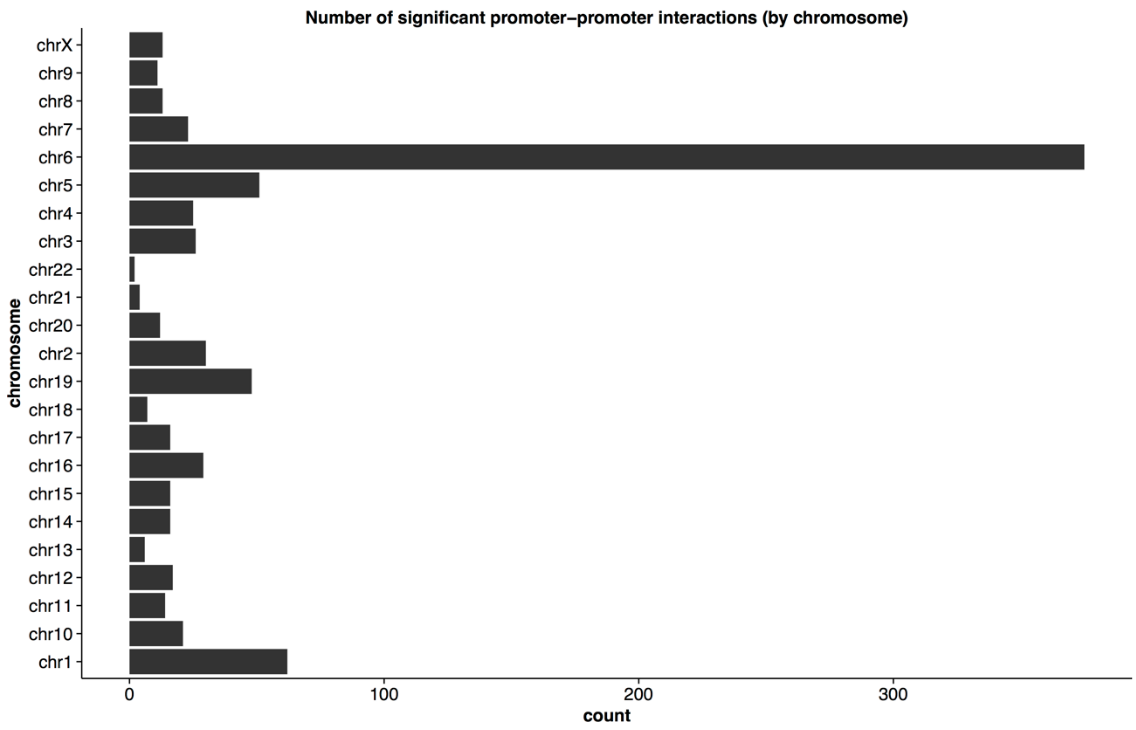

Supplementary Figure 31 – Absolute number of significant promoter-promoter interactions
Significant HiC-DC interactions (FDR < 1%) for the Rao et al.[1] GM12878 data annotated as promoter-promoter contacts per the taxonomy in **Supplementary Figure 24**. The vast majority of contacts are located on chromosome 6.

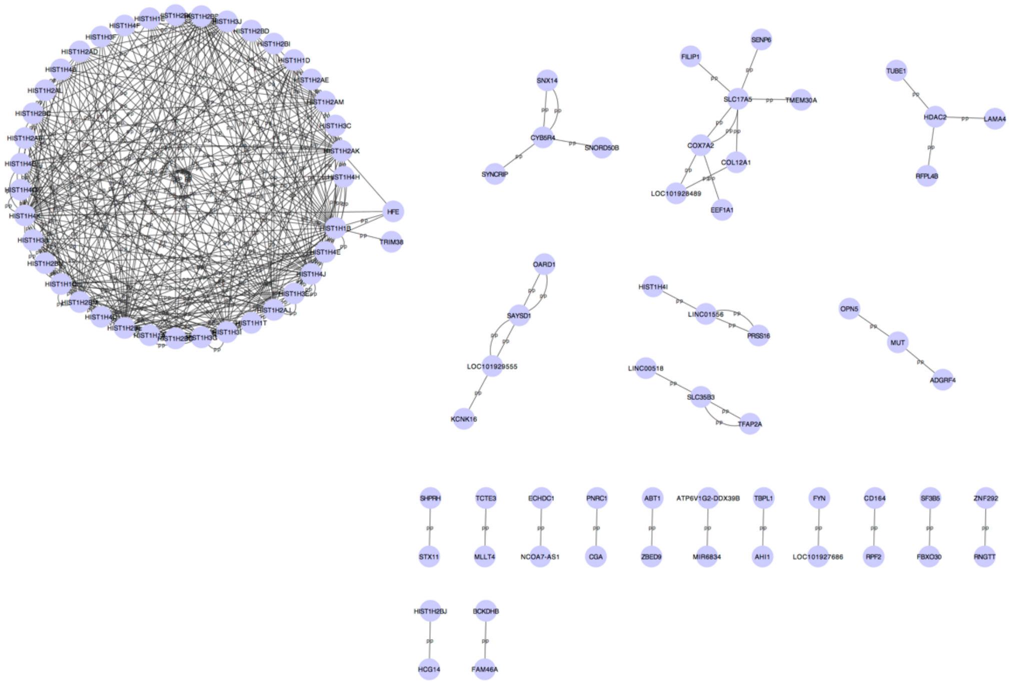

Supplementary Figure 32 – Network visualization of significant contacts and their associated genes on chromosome 6

Significant HiC-DC interactions (FDR < 1%) for the Rao et al.[1] data annotated as promoter-promoter contacts that are located on chromosome 6, visualized as a graph using Cytoscape. Each node is a gene, and each edge is a Hi-C contact that connects two genes. The histone genes appear in both the largest and most densely connected subgraph.

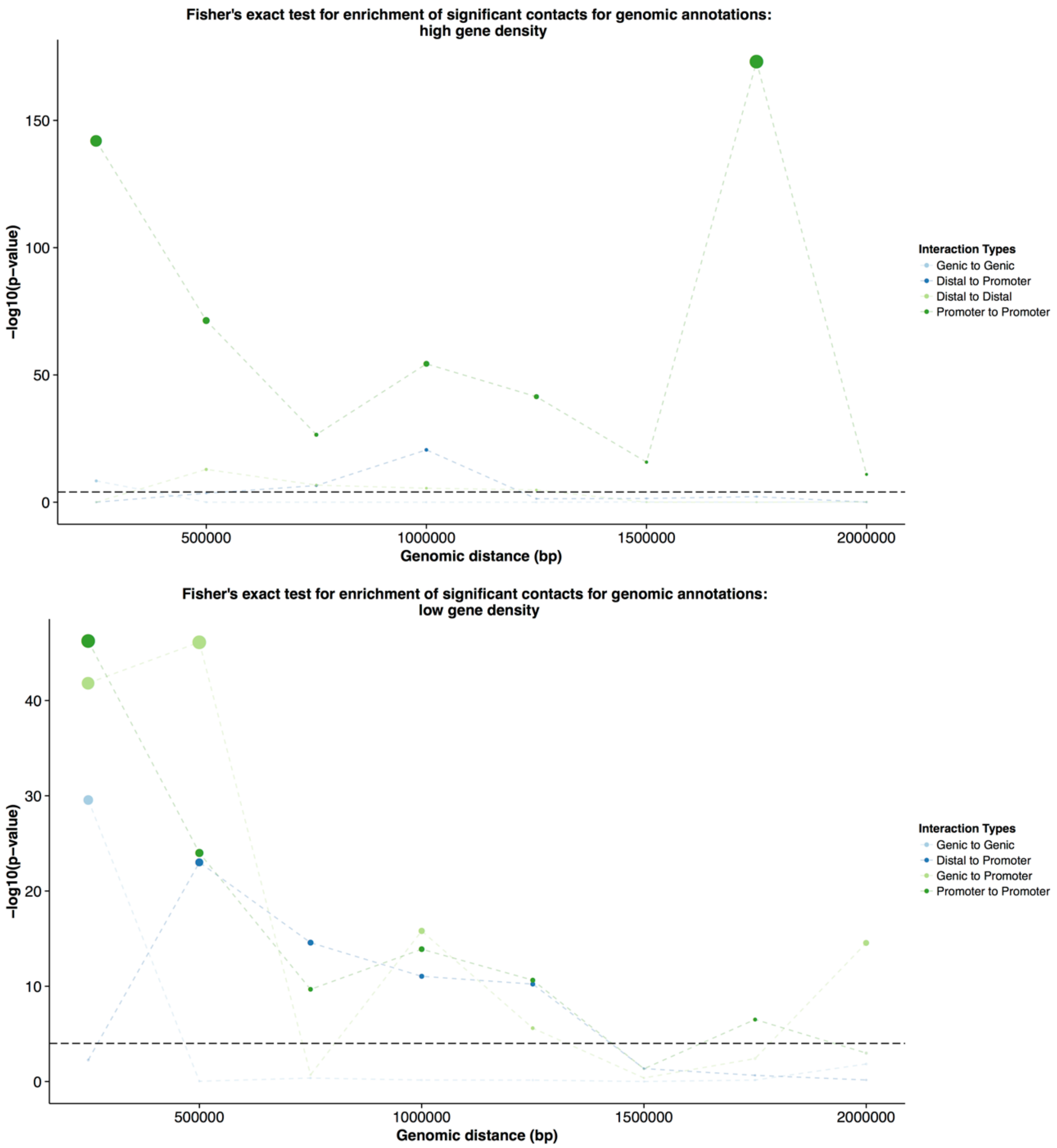

Supplementary Figure 33 – Gene density analysis of promoter-promoter contacts

Enrichments of significant (FDR < 1%) HiC-DC interactions annotated by genomic location (see **Figure 3b**) for 250kb regions for the Rao et al.[1] data by Wilxocon rank-sum test. The contacts were subdivided into high and low gene-density regions according to the average of the gene densities of both genomic bin endpoints (see **Supplementary Figure 36**). Promoter-promoter contacts are enriched in the (1.5Mb – 2Mb) range strongly within high-density regions, yet only mildly within low-density regions. Gene-promoter contacts are significantly enriched within low-density regions in the (0-500kb) range, as were the distal-promoter contacts, yet not within the high-density regions.

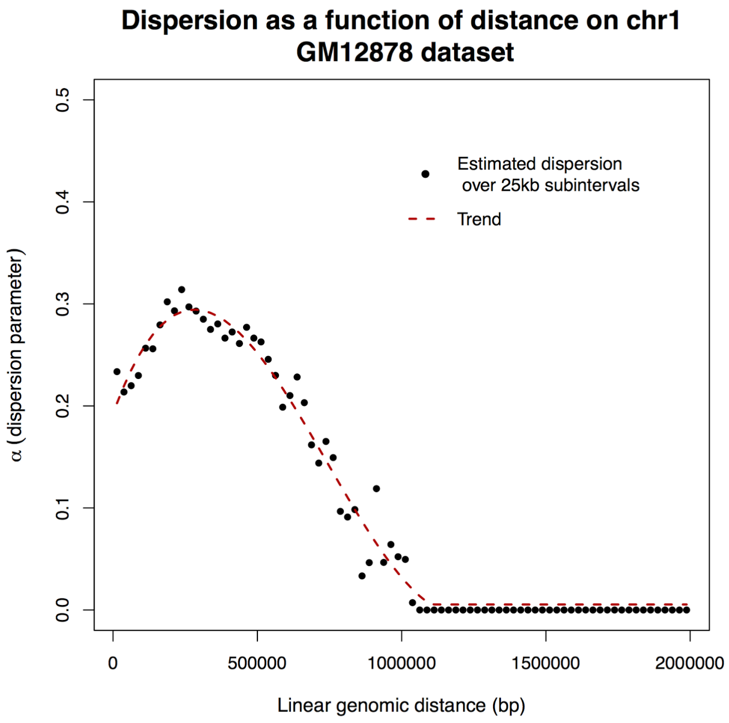

Supplementary Figure 34 – Dispersion parameter as a function of genomic distance
We partitioned genomic distance into 25kb intervals and trained HiC-DC on bin count data from each interval for the Rao et al.[1] GM12878 (primary replicate) data for chromosome 1, therefore estimating a separate dispersion parameter for each interval. Our analysis showed that the dispersion parameter does vary with genomic distance, and for linear genomic distance > 1 Mb, the estimated dispersion parameter is close to zero. This suggests that bin counts for longer-range contacts approximately follow a Poisson regression model.

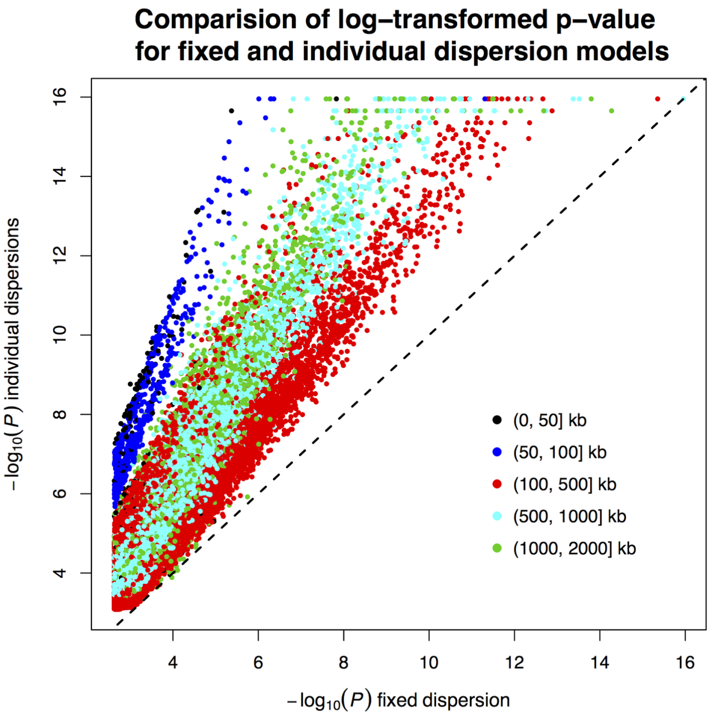

Supplementary Figure 35 – Scatterplot of $-\log_{10}$ *P*-values of significant interactions called by HiC-DC for individual dispersion parameters versus $-\log_{10}$ *P*-values of significant interactions called by HiC-DC for constant dispersion stratified by distance

We partitioned genomic distance into 25kb intervals and trained a separate HiC-DC model (with bin-dependent estimate of the dispersion parameter) on each interval for the Rao et al.[1] GM12878 (primary replicate) data on chromosome 1. We used the corresponding models to assign *P* values to bins within each distance band. We also trained HiC-DC on the whole dataset, using a single dispersion parameter in the model. The results show that interactions identified as significant by HiC-DC using a fixed dispersion parameter would also be called by HiC-DC models with distance-specific dispersion parameters.

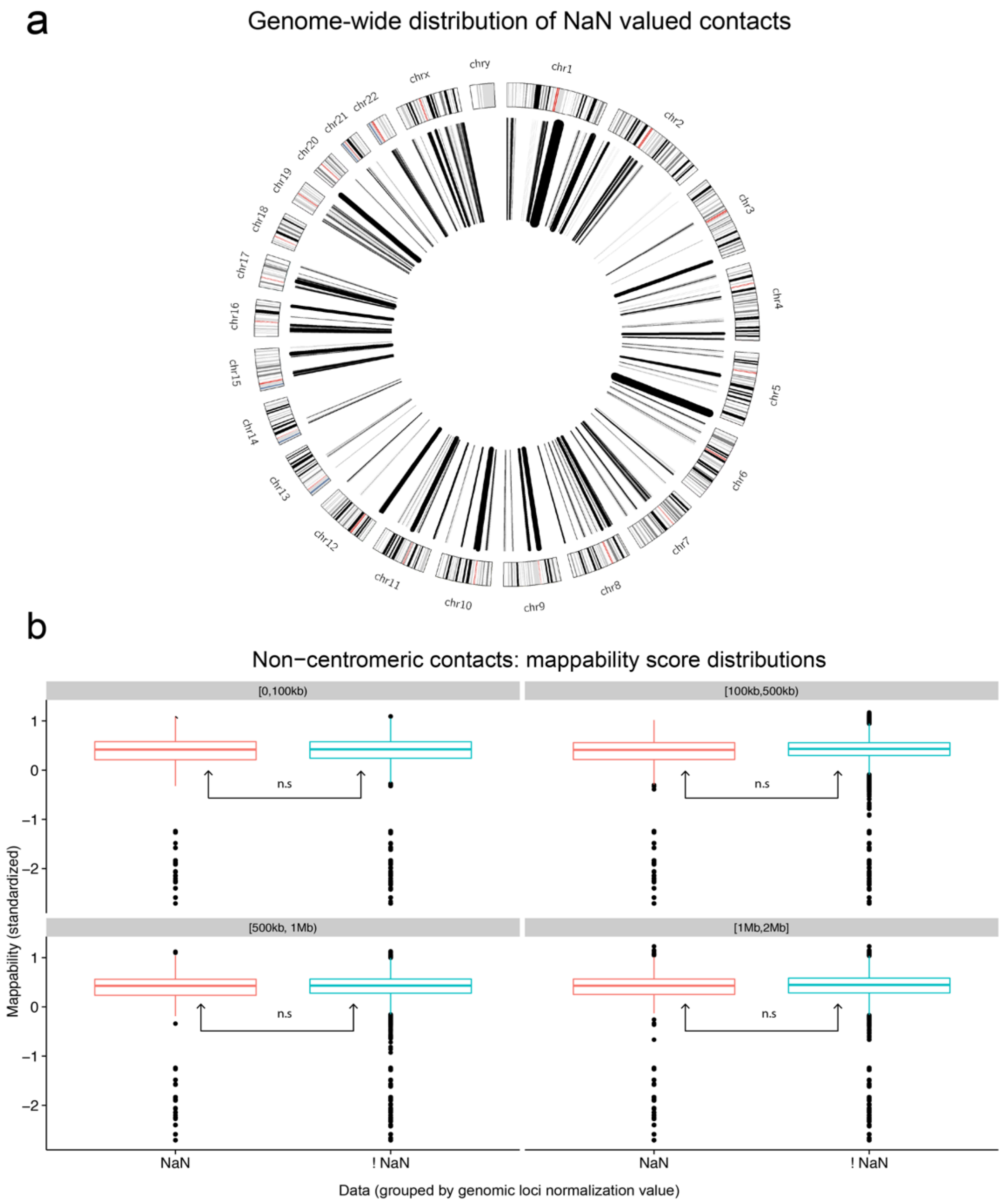

Supplementary Figure 36 – Contacts lost to NaN normalization factor values for ICE
(a) 12,353 (494 with FDR < 1%) contacts with non-centromeric genomic endpoints that were excluded from Fit-Hi-C by virtue of one or both genomic bins having been assigned the value NaN. To ensure numerical stability, ICE removes certain rows and columns from the count matrix prior to normalization, resulting in substantial loss of interactions. While the distribution of genomic regions that are linked by those contacts assigned NaN normalization values is concentrated near centromeric or telomeric regions, 95 percent (12,353 of a total 12,919) lie within non-centromeric and non-telomeric regions on most chromosomes. **(b)** Standardized read mappability scores of the genomic bins involved in the contacts displayed in (a) versus a similarly sized random sample of contacts genome-wide. We observed no significant difference in mappability scores.

Chr	start I (kb)	end I (kb)	start J (kb)	end J (kb)	start K (kb)	end K (kb)	Smallest significant P value	Significant contacts	Control contacts	Smallest control P value	Largest significant P value	Largest control P value
11	13072	13075	13029	13032	12986	12989	0	54	110	1	0.009	1
13	8637	8640	8546	8549	8455	8458	0	32	70	1	0.007	1
14	7160	7163	7220	7223	7280	7283	0	46	80	0.320	0.008	1
17	6676	6679	6722	6725	6768	6771	0	70	56	1	0.009	1

Supplementary Table 1 – HiC-DC analysis of FISH loci from Rao et al.[1]
Validation of HiC-DC on loci identified from FISH experiments performed by Rao et al.[1]. We took four loci identified by FISH as true positive interactions (in this table as I – J), as well as four loci chosen by Rao et al.[1] as true negative interactions (in this table as J-K), and computed the significance for all contacts in the Rao et al.[1] GM12878 data set (5kb fixed binning) which spanned bins I to J (positive interactions) and bins J to K (control interactions). We observed that over all four FISH experiments, HiC-DC identifies many significant contacts connecting the true positive loci (I-J) and does not identify any significant contacts connecting the control loci (J-K). We report the most and least significant *P* values for each pair of FISH loci

Supplementary References

1. Rao SS*, et al.* A 3D map of the human genome at kilobase resolution reveals principles of chromatin looping. *Cell* **159**, 1665-1680 (2014).
2. Dixon JR*, et al.* Topological domains in mammalian genomes identified by analysis of chromatin interactions. *Nature* **485**, 376-380 (2012).
