# Supplementary material for: An integrated model for detecting significant chromatin interactions from high-resolution Hi-C data
Source: Nat Commun. 2017 May 17;8:15454. doi: 10.1038/ncomms15454 (PMC5442359; doi:10.1038/ncomms15454)
Supplement: Supplementary Software 1 — HiC-DC R package [file ncomms15454-s3.zip › hic.dc/inst/doc/Vignette.html]

An Introduction to HiC-DC


# An Introduction to HiC-DC

#### *Mark A. Carty and Merve Sahin*

#### *2/28/2017*

## Introduction

HiC-DC package provides tools for data processing, estimating the statistical significance of chromatin interactions, and visualizing chromosomal contact maps produced by Hi-C. HiC-DC tests for chromatin interactions by using a zero-inflated negative binomial generalized linear model. This vignette explains the use of the package and demonstrates typical workflows.

## Input Data

As input, the HiC-DC begins with bam files obtained from a Hi-C sequencing experiment. **FilterHi-C** parses two bam files for data curation and produces a *GenomicRanges* object of paired-end reads. Reading the associated records from the BAM files is accomplished as follows:

```
library(HiC.DC)
```

```
## Warning: replacing previous import 'GenomicRanges::shift' by
## 'data.table::shift' when loading 'HiC.DC'
```

```
options(warn=-1)

#find the location of the data folder
path.data=system.file("data", package="HiC.DC")

genome = 'Hsapiens'                                                          # Genome
version = 'hg19'                                                             # Version of build
bamfiles1 = paste0(path.data,"/602_1_chr21_little.bam")
bamfiles2 = paste0(path.data,"/602_2_chr21_little.bam")

         # Hi-C Bam files
chr = 'chr21'                                                                # chromosome
cores = 1                                                                    # Number of threads
outputfile = paste0(path.data,"/602_chr21_little.rds")
         # Out put file

filterHiC(genome, version, bamfiles1, bamfiles2, outputfile, chr, cores)
```

```
## Using chr21 for analysis 
## Registering 1 cores for parallel processing 
## [1] "Time required to get reads: 0.30 mins"
```

As a sanity check, we load the RDS file with the *GenomicRanges* object of paired-end reads into R.

```
data = readRDS(outputfile)
data
```

```
## GRangesList object of length 2:
## $R1 
## GRanges object with 52 ranges and 0 metadata columns:
##        seqnames             ranges strand
##           <Rle>          <IRanges>  <Rle>
##    [1]    chr21 [9429616, 9429711]      -
##    [2]    chr21 [9429161, 9429234]      +
##    [3]    chr21 [9413769, 9413864]      +
##    [4]    chr21 [9432035, 9432130]      +
##    [5]    chr21 [9417188, 9417283]      -
##    ...      ...                ...    ...
##   [48]    chr21 [9423432, 9423527]      +
##   [49]    chr21 [9432875, 9432970]      +
##   [50]    chr21 [9429971, 9430066]      -
##   [51]    chr21 [9413782, 9413838]      -
##   [52]    chr21 [9432124, 9432219]      -
## 
## ...
## <1 more element>
## -------
## seqinfo: 1 sequence from an unspecified genome
```

## Create Genomic Bins

Since we successfully created our *GenomicRanges* object of paired-end reads, let us make another *GenomicRanges* object of genomic intervals or bins for chromosome 21 based on the restriction cutting sites for that chromosome. We will use **Make\_bins** to create the intervals. The arguments to **Make\_bins** include the genome, the build, the restriction enzyme motif, the bin type, the number of restriction cutting sites aggregated within a bin (e.g. use the argument “numsites”), the output path, the filename, and the chromosome. If you wanted to use uniform binning, replace the argument “numsite” with “binsize.”

```
path = paste0(path.data,'/chr21')
fname = paste0(path.data,'/wgEncodeCrgMapabilityAlign50mer_chr21.bigWig')
chr = 'chr21' 
genome = 'Hsapiens'                                                          
version = 'hg19'  

Make_Bins(genom = genome, genom.version = version, bin.type = 'Bins-uniform', sig = 'GATC', 
          binsize = 50e3, output.path = path, con = fname, chrom = chr)
```

## Generate Hi-C Counts

We have all the data required to produce Hi-C counts. Now, let us use runhic to generate Hi-C count data. You must provide the following RDS files to use runhic: the path to read, bin, enzyme cut sites file locations. Below is an example of how to use **runhic** to generate Hi-C counts:

```
read.file = paste0(path.data,'/602_chr21_little.rds')
bin.file = paste0(path.data,'/chr21_binsGR.rds')
enzyme.file = paste0(path.data,'/chr21_enzymeCuts.rds')
output.file = paste0(path.data,'/HiC_Counts_chr21.rds')

runhic(genome, version, readfile = read.file, 
       binfile = bin.file, REfile = enzyme.file, outfile = output.file,
       chrom = chr)
```

```
## Using chr21 for analysis 
## [1] "chr21 took 0.34 seconds"
## Registering 1 cores for parallel processing 
## [1] "Time 12.54 seconds"
## Registering 1 cores for parallel processing 
## [1] "Time require to get counts 13.54 seconds"
```

```
##           used  (Mb) gc trigger  (Mb) max used  (Mb)
## Ncells 5644773 301.5    8273852 441.9  5644773 301.5
## Vcells 3760251  28.7   11412464  87.1  3760251  28.7
```

## Generate Features

We create the model matrix used by the hurdle negative binomial generalized model. We must provide the path to the directory of the Hi-C count RDS files and the identifier for those files. The function **Make\_Features** creates the model matrix:

```
path = path.data
file.prefix = 'HiC_Counts'
output = paste0(path.data,'/HiC_data')

Make_Features(path, file.prefix, bin.file, chr, 'Bins-uniform', output = output)
```

```
##           used  (Mb) gc trigger  (Mb) max used  (Mb)
## Ncells 5646642 301.6    8273852 441.9  5646642 301.6
## Vcells 3761390  28.7   11412464  87.1  3761390  28.7
```

## Run HiC-DC to Test for Chromatin Interactions

Before we run **hic.dc** on a dataset, we want to determine the appropriate subsample size to train the model. HiC-DC takes a subsample of the data from the model matrix, which is data.table, for training. We need to have enough zero-count events to fit the hurdle negative binomial regression. We should consider several different subsamples sizes for training to determine the size that ensures that HiC-DC runs efficiently.

To test for chromatin interactions from Hi-C sequencing data, we can run either **hic.dc** or **hic.dc.fixedbin** as follows:

```
file = paste0(path.data,'/HiC_data_Features_80kb_on_chr12.rds')

df = 6
ssize = 0.8
binsize = 8e4
chunksize = 1.4e4
output = paste0(path.data,'/HiC_data')
hic.dc.fixedbin(file = file, df = df, ssize = ssize, cores = cores, binsize = binsize, output = output, chunksize = chunksize)
```

```
## [1] "Time required to fit the model: 0.01 mins"
## [1] "Time required to estimate additional parameters : 0.25 mins"
```

```
##           used  (Mb) gc trigger  (Mb) max used  (Mb)
## Ncells 5676775 303.2    8273852 441.9  5676775 303.2
## Vcells 3806634  29.1   11412464  87.1  3806634  29.1
```

```
system(paste0("rm ",path.data,"/HiC_data_Model-Fit_on_chr12.rds"))
```

## HiC-DC Results

Let us look at the significant interactions beyond the diagonal detected at 5% FDR threshold from the **hic.dc** run:

```
data = readRDS(paste0(path.data,"/HiC_data_Results_on_chr12.rds"))
signif.contacts = data[qvalue < 0.05 & D!=0, list(binI=binI, binJ=binJ, 
                         counts=counts, qvalue=qvalue)]
head(signif.contacts)
```

```
##                         binI                      binJ counts       qvalue
## 1: chr12-115040001-115120000 chr12-116320001-116400000   2616 0.000000e+00
## 2:   chr12-91760001-91840000   chr12-92960001-93040000   2656 0.000000e+00
## 3:   chr12-10880001-10960000   chr12-11600001-11680000   2805 3.576508e-02
## 4:   chr12-92160001-92240000   chr12-92960001-93040000   3096 1.452931e-06
## 5: chr12-104720001-104800000 chr12-105440001-105520000   3527 2.046620e-02
## 6: chr12-102320001-102400000 chr12-103120001-103200000   3532 1.452931e-06
```

## Visualization of Hi-C Contacts with Epigenetic and Genomic Elements

We plot Hi-C hotspots estimated by **hic.dic** along with epigenetic tracks for GM12878 showing DNase I hypersensitive sites, H3K27ac, ChIP-seq, and RAD21.

```
start = 11500000
end = 11840000
size = 1e5
col = c("red","blue","darkgreen" ,"brown")

regions = readRDS(paste0(path.data,'/chr10_binsGR.rds'))
trackslist = readRDS(paste0(path.data,'/trackslist.rds'))
contact.matrix = readRDS(paste0(path.data,'/HiC_data_Results_on_chr10_20kb.rds'))

Signaltracks_hic(contact.matrix = contact.matrix, regions = regions,
                 start=start, end=end, trackslist=trackslist, 
                 size = size, col=col)
```

We have a Sashimi plot depiction of significant interactions called by **hic.dc** showing chromatin looping between the hotspots seen in plot above.

```
sashimi.plot(contact.matrix = contact.matrix, chromstart = start, 
             chromend = end, regions = regions)
```
